# FOXO3 regulates a common genomic program in aging and glioblastoma stem cells

**DOI:** 10.1002/aac2.12043

**Published:** 2021-12-18

**Authors:** Amanda J. Audesse, Galina Karashchuk, Zachary A. Gardell, Nelli S. Lakis, Sun Y. Maybury-Lewis, Abigail K. Brown, Dena S. Leeman, Yee Voan Teo, Nicola Neretti, Douglas C. Anthony, Alexander S. Brodsky, Ashley E. Webb

**Affiliations:** 1 Neuroscience Graduate Program, Brown University, Providence, Rhode Island, USA; 2 Department of Molecular Biology, Cell Biology, and Biochemistry, Brown University, Providence, Rhode Island, USA; 3 Department of Pathology and Laboratory Medicine, Lifespan Academic Medical Center and Warren Alpert Medical School at Brown University, Providence, Rhode Island, USA; 4 Department of Pathology and Laboratory Medicine, University of Kansas Medical Center, Kansas City, Kansas, USA; 5 Molecular Biology, Cell Biology, and Biochemistry Graduate Program, Brown University, Providence, Rhode Island, USA; 6 Department of Discovery Immunology, Genentech, Inc., South San Francisco, California, USA; 7 Center on the Biology of Aging, Brown University, Providence, Rhode Island, USA; 8 Department of Neurology, Brown University, Providence, Rhode Island, USA; 9 Carney Institute for Brain Science, Brown University, Providence, Rhode Island, USA

**Keywords:** aging, glioma, stem cells, transcriptomics

## Abstract

**Background:**

Glioblastoma (GBM) is an aggressive, age-associated malignant glioma that contains populations of cancer stem cells. These glioma stem cells (GSCs) evade therapeutic interventions and repopulate tumors due to their existence in a slowly cycling quiescent state. Although aging is well known to increase cancer initiation, the extent to which the mechanisms supporting GSC tumorigenicity are related to physiological aging remains unknown.

**Aims:**

Here, we investigate the transcriptional mechanisms by which Forkhead Box O3 (FOXO3), a transcriptional regulator that promotes healthy aging, affects GSC function and the extent to which FOXO3 transcriptional networks are dysregulated in aging and GBM.

**Methods and results:**

We performed transcriptome analysis of clinical GBM tumors and observed that high FOXO3 activity is associated with gene expression signatures of stem cell quiescence, reduced oxidative metabolism, and improved patient outcomes. Consistent with these findings, we show that elevated FOXO3 activity significantly reduces the proliferation of GBM-derived GSCs. Using RNA-seq, we find that functional ablation of FOXO3 in GSCs rewires the transcriptional circuitry associated with metabolism, epigenetic stability, quiescence, and differentiation. Since FOXO3 has been implicated in healthy aging, we then investigated the extent to which it regulates common transcriptional programs in aging neural stem cells (NSCs) and GSCs. We uncover a shared transcriptional program and, most strikingly, find that FOXO3-regulated pathways are associated with altered mitochondrial functions in both aging and GBM.

**Conclusions:**

This work identifies a FOXO-associated transcriptional program that correlates between GSCs and aging NSCs and is enriched for metabolic and stemness pathways connected with GBM and aging.

## 1 | INTRODUCTION

Glioblastoma (GBM, formerly glioblastoma multiforme) is a grade IV astrocytoma, and one of the most lethal human cancers. The incidence increases with age, preferentially affecting adults over 55 years of age, with a median survival of 15–18 months. Despite multifaceted therapies, the 5-year survival rate is only 5%.^1,2^ The median age of onset in isocitrate dehydrogenase (IDH)-wildtype GBM is over 60; these tumors do not have tumorigenic mutations in either of the IDH genes (*IDH1*, *IDH2*).^2,3^ The mechanisms responsible for increased incidence with age are not fully understood but include accumulation of genomic damage, altered epigenetic states, and changes to the microenvironment.

GBMs are heterogeneous tumors and contain populations of treatment-resistant stem-like cells termed glioma stem cells (GSCs).^4–7^ Like somatic stem cells, GSCs have the capacity to self-renew and differentiate. With age, somatic stem cells undergo functional decline due to altered metabolism, increased DNA damage, defective proteostasis, and epigenetic drift.^8,9^ Similar alterations occur in the context of cancer, but the extent to which cancer stem cells and aged stem cells share specific features is not well known. Moreover, whether common molecular regulators preserve healthy stem cell function and prevent oncogenic transformation is not understood.

GSCs share phenotypic characteristics with endogenous neural stem cells (NSCs), including quiescence, episodic proliferation, and multipotency. Evidence from rodents indicates that the majority of endogenous NSCs are in a reversible state of cell cycle arrest termed quiescence. In response to extrinsic or intrinsic cues, quiescent NSCs can re-enter the cell cycle and undergo a short burst of proliferation. Proliferating NSCs can either re-enter quiescence or differentiate into neurons or glia. Similarly, treatment-resistant quiescent GSCs have been identified that eventually give rise to highly proliferative cells and establish secondary tumors.^10,11^ Conventional treatments that target the highly proliferative populations within the heterogeneous tumor environment only transiently eliminate the tumor in most GBM cases and can promote a stem-like state.^5,12,13^ However, it remains unclear how dysregulation of normal NSC properties, which occur during the aging process, contribute to GBM pathogenesis and resistance to treatment.

Several lines of evidence suggest that GSCs can hijack normal neurogenic programs to promote the maintenance of a stem-like state. For example, activation of transcription factor networks such as SOX2, OLIG2, HEY1, and ASCL1 are enriched in GSCs, compared to differentiated cells.^14^ SOX2, in particular, has been linked to stemness in healthy NSCs and its loss is associated with impaired neurogenesis and depletion of the functional stem cell pool in adult mice.^15,16^ SOX2 is highly expressed in GBM tumor samples and is associated with malignancy and tumor-propagating capacity in gliomas.^17–24^ Further, SOX2 is enriched in undifferentiated and proliferating cells in GBM and is vital for the stemness of these populations, again reinforcing a functional role for endogenous NSC networks in GSCs.^17,25–27^

The key transcriptional networks that maintain quiescent GSCs and endow them with chemo- and radioresistance are not known. In endogenous NSCs, the pro-longevity transcription factor and tumor suppressor, FOXO3, functions to promote quiescence and restrain a proliferative transcriptional program.^28–30^ In adult mammalian NSCs, FOXO3 regulates *p27/kip1,* which promotes cell cycle arrest in a two-pronged mechanism as both a regulator of quiescence and cell cycle exit in immature neurons.^31^ Moreover, altered FOXO signaling has been linked to GBM tumorigenesis, with low FOXO3 levels correlating with poor outcomes.^32^ FOXOs can function as tumor suppressors in different contexts,^33–36^ and in GBM cells, FOXO3 activation restricts tumor growth.^37^ More generally, the PTEN/AKT/FOXO (phosphatase and tensin homolog/AKT serine/threonine kinase/FOXO) pathway is one of the most commonly mutated pathways in GBM; more than half of GBMs harbor mutations in PTEN, which functions upstream of FOXO3.^38–40^ Notably, one study found that high FOXO3 levels, particularly when combined with other markers, was associated with lower overall survival in GBM,^41^ underscoring the need to better understand FOXO3’s role in GBM. FOXO3 has also been broadly implicated in healthy aging. FOXOs promote longevity through the maintenance of cellular homeostasis under changing environmental conditions such as stress or altered nutrient availability. Through these actions, FOXOs preserve stem cell pools in vivo, including in the brain. The extent to which FOXO3’s pro-longevity functions overlap with its tumor-suppressive mechanisms is not known.

Here, we identify transcriptional signatures associated with overall *FOXO3* transcript levels in GBM, including metabolic networks and features of stem cell dormancy. Interestingly, we observe that the signatures associated with high *FOXO3* levels correlate with improved survival early in the clinical course. We also identify the transcriptional networks that are altered by FOXO3 ablation in patient-derived GSCs and aged endogenous NSCs. We find that these FOXO3-associated transcriptional networks correlate in GSCs and old NSCs with a particular enrichment for metabolic pathways, including mitochondrial functions, which have also recently been identified in a pathway-based subtype of GBM.^42^ Overall, we find that FOXO3 shares a common transcriptional network in aging and cancer that is associated with the stabilization of metabolic states.

## 2 | METHODS

### 2.1 | The Cancer Genome Atlas Project (TCGA) RNA-seq analysis

Bulk tumor RNA-seq and associated clinical and meta-data were obtained from TCGA. GBM tumor samples were downloaded using the GDC Data Transfer Tool and GDC data portal from the National Cancer Institute. HTSeq–FPKM (fragments per kilobase per million) files were downloaded per the data manifest for GBM. For TCGA GBM, there were 174 samples available, including five normal samples. After performing a log2(FPKM + 1) on all FPKM values, normal samples were removed, and the remaining samples were split according to expression quartile for *SOX2* and/or *FOXO3*. To identify tumors with high or low *FOXO3* expression, GBM samples in the top and bottom quartiles were selected for analysis and termed as *FOXO3* high and *FOXO3* low (42 GBMs per group). To identify GBMs with high or low *FOXO3* and *SOX2*, samples in the top or bottom quartile of expression for both factors were selected (21 and 17 GBMs, respectively). To obtain sufficient samples for survival and subtype analysis, the samples were split by median expression, with 51 GBMs per group. Samples that fell above the median expression for one factor and below the median expression of the other factor were excluded in all analyses.

### 2.2 | Ivy GBM Atlas Project (Ivy GAP) RNA-seq analysis

RNA-seq data were downloaded from the Ivy GAP. The dataset included 148 RNA-seq samples from 34 tumors. Cancer stem cell clusters were identified by in-situ hybridization with an 18 probe reference set. As stated in the documentation, FPKM values were normalized across all samples based on genes not enriched in the selected anatomic structures.^43^ After performing a log2(FPKM + 1) on all FPKM values, files were split according to expression quartile for *FOXO3* and termed *FOXO3* high (23 tumor samples) and *FOXO3* low(22 tumor samples). Samples that were collected based on the low expression of reference genes (CT-controls) were not considered in the analysis unless stated otherwise.

### 2.3 | Analysis of FOXO3 RNA and protein levels in GBM

cBioPortal was used to investigate the relationship between FOXO3 at the RNA and protein levels, utilizing processed publicly available TCGA GBM data by Brennan et al.^19^ GBM tumor samples were grouped by quartile in the cBioPortal based on *FOXO3* transcript level (RNAseq V2 RSEM). FOXO3 protein levels (RPPA) were then visualized between groups of GBM tumor samples. A total of 70 cases had both RNA and protein-level data available. FOXO3 RPPA values for each group were plotted using Prism.

### 2.4 | Single sample gene set enrichment analysis (ssGSEA)

For ssGSEA analysis of TCGA GBM and Ivy GAP, datasets were formatted as gct files and uploaded to GenePattern.^44,45^ The ssGSEA projection module was run against all gene ontology (GO) categories (c5.all). Default settings were used, and the sample normalization method was specified as“log.” Enrichment scores were determined for each sample and GO category, followed by differential expression analysis to determine the categories that were differentially up- or downregulated between *FOXO3* high and *FOXO3* low samples in the FOXO3 analysis or *FOXO3/SOX2* high and low samples for the shared neural progenitor cell (NPC) network analysis. The limma package was used to perform differential expression analysis in RStudio. The Gene Set Variation Analysis (GSVA) vignette from Bioconductor was used to guide this analysis. For the *FOXO3* analysis in TCGA GBM samples, enrichment scores were considered significant with an adjusted *p*-value cutoff of *p* < 0.0001 following a Benjamini–Hochberg correction. The top 300 GO categories according to differential expression analysis are displayed as a heatmap with ssGSEA enrichment scores scaled by row. For the *FOXO3* analysis in Ivy GAP samples, enrichment scores were considered significant with an adjusted *p*-value cutoff of *p* < 0.04 following a Benjamini–Hochberg correction. All significant categories according to differential expression analysis are displayed as a heatmap with ssGSEA enrichment scores scaled by row. For the analysis of *FOXO3/SOX2* high and low samples, the top 10 most significantly up- and downregulated categories identified by differential expression analysis are displayed as log2(fold change) (LFC) ssGSEA enrichment score. For the construction of the heatmaps, Pretty Heatmaps (pheatmaps, version 1.0.12) was implemented in RStudio.

### 2.5 | Upstream regulator analysis

Epigenetic landscape in silico deletion analysis (LISA) was used to identify putative transcriptional regulators.^46^ The input gene set for the genes differentially expressed between *FOXO3* high and *FOXO3* low GBMs included genes with a LFC cutoff of 0.8 and adjusted *p*-value cutoff of *p* < 0.01.

### 2.6 | Identification of a shared gene regulatory network between SOX2 and FOXO3

The FOXO3 direct targets were previously identified by chromatin immunoprecipitation followed by direct sequencing (ChIP-seq) in adult mouse neural stem and progenitor cells.^30,47^ To allow the direct comparison of FOXO3 and SOX2 gene networks, FOXO3 targets were converted to human gene symbols using the Mouse Genome Informatics ortholog table (informatics.jax.org). SOX2 ChIP-seq performed in human NPCs (hNPCs) raw sequencing data files were obtained from the Gene Expression Omnibus repository (SRR2049336, SRR2049338 SOX2 ChIP, and input samples). Reads were aligned to the hg19 reference genome using Hisat2 (version 2.1.0). Duplicate reads were marked with Picard (version 2.9.2) and removed with SAMtools (version 1.9). MACS2 (version 2.1.1) was used to call peaks with a *q* value of 0.001. Peaks were assigned to genes using the Genomic Regions Enrichment of Annotations Tool (GREAT), with the parameters to −25 kb/+10 kb around the transcription start sites. Venn diagrams were created using Vennerable (version 3.0) in RStudio. Statistical enrichment analysis was performed using Fisher’s exact test using all unique human gene symbols as background. For the Oligodendrocyte transcription factor 2 (OLIG2) analysis, targets from the publicly available dataset GSM2112627^48^ were downloaded from the CistromeDB^49^ (ID 69350).

### 2.7 | GO analysis

The PANTHER database was used to identify statistically over-represented biological process GOs (complete biological process). The default human whole-genome list was used as background. Test type was indicated as binomial with the Bonferroni correction for multiple testing. Results were then grouped by the hierarchical organization indicated in the PANTHER output, where the most specific subclass is listed first with the parent terms indicated below and ordered according to the corrected *p*-value.^50^

GSEA Preranked analysis (version 6.0.12) was performed to determine enrichment against all GO categories (C5.all.V6.symbols.gmt) or custom NSC signatures using GenePattern.^44^ Otherwise, default settings were used and GO categories with Familywise-error rate (FWER)-corrected *p-values* < 0.05 were considered significant enrichments. Ranked lists were based on LFC values, with limma being used to identify the differential expression values at the gene level.

### 2.8 | Stemness index and subtype analyses

Molecular subtype classifications of TCGA GBM data were reclassified from the original specifications.^51^ This new GBM-instrinsic transcriptional subtype was used to classify tumors into three categories: mesenchymal, classical, and proneural.^52^ We mapped these classifications to our different GBM tumor sample populations from TCGA using the available RNAseq file names where data were available. Stemness indices reflective of epigenetic features (mDNAsi) and gene expression features (mRNAsi) were obtained by Malta et al. who define and quantify the degree of oncogenic dedifferentiation of tumor samples.^53^ To obtain an independent quantification of stemness of our different GBM tumor sample populations from TCGA, we used the available patient ID to obtain the corresponding mRNAsi values for each of our TCGA GBM samples.

### 2.9 | Prism

Bar graphs and violin plots were generated using Graphpad Prism 8.0.

### 2.10 | Signatures of activated and quiescent NSCs

Custom signatures representing the activated and quiescent NSC populations were generated using three independent datasets.^54–56^ Each study reported differential expression results between endogenous activated and quiescent NSC populations in the mouse. In the case of the Dulken et al. data, the differential expression results between the quiescent and the early activated NSC populations were used. Results from each analysis were ordered by adjusted fold change values or *z*-scores and the top 500 genes that were upregulated in either the quiescent or activated NSCs were selected. Genes that were present among the top 500 in two/three datasets were used in GSEA preranked analysis as the quiescent and activated signatures. Default settings were used and GO categories with FWER-corrected *p*-values < 0.05 were considered significant enrichments.

### 2.11 | Signatures of FOXO3 transcriptional activity in NSCs

Custom signatures representing the gene signatures of FOXO3 activation and FOXO3 repression were generated using the top 200 differentially expressed genes from the aged quiescent or activated NSC mouse datasets, comparing the wild type to *Foxo3* null NSCs. The aged quiescent transcriptional signature of FOXO3-repression was used to correlate FOXO signatures with FOXO3 levels in GBM since this was the most affected cell type following ablation of *Foxo3*. After converting the gene signatures to human gene names, ssGSEA was used to calculate an enrichment score of FOXO3 repression for each individual GBM tumor sample and the resulting ssGSEA enrichment scores and corresponding FOXO3 expression were analyzed using linear regression analyses in Prism. GBM tumor samples with high enrichment (top quartile of ssGSEA enrichment scores) or low enrichment (bottom quartile of ssGSEA enrichment scores) for the signature of FOXO3 repression were run with ssGSEA projection and enriched GO categories were identified as described above in the detailed methods for ssGSEA analysis (with corrected *p*-values of *p* < 0.00001).

### 2.12 | Immunohistochemistry in GBM tissue microarrays (TMAs)

Cases of GBM from the Rhode Island and the Miriam Hospitals from January 1, 2012, through August 31, 2016, were retrieved by searching through the pathology database for “glioblastoma” or “gliosarcoma”; a total of 270 cases were identified. Histologic diagnosis was confirmed by two neuropathologists (NSL, DCA) and only those cases that fulfilled the diagnosis of GBM according to the 2016 World Health Organization (WHO) classification^2,3^ were included. Also required for inclusion was clinical follow-up information and sufficient tissue remaining in the diagnostic block to create a TMA. If the original block used for diagnosis and immunohistochemistry was no longer available, a second appropriate block was chosen. The two neuropathologists (NSL, DCA) agreed on the diagnoses of all tumor samples using the WHO criteria.^2,3^

#### 2.12.1 | TMA

A total of 98 cases had sufficient tumor remaining in the block to be included in a deidentified paraffin-embedded TMA. Eighty-two cases had three representative tumor regions only, and 16 cases had three representative tumor regions plus a “normal” or “infiltrating edge of tumor.” The tissue cores were evaluated by two neuropathologists (NSL, DCA) who supervised all TMA construction steps. From the TMA blocks, 4-*μ*m thick sections were prepared. Individual cores were classified as “tumor,” “normal brain,” or “insufficient tissue/completely necrotic/missing core.” Cores were not included in the final analysis if the core was lost, severely damaged, and/or did not have the highly cellular tumor on the tumor cores. Nine cases were excluded due to the lack of any analyzable tumor in the final TMA blocks or because they were recurrent tumors that did not meet diagnostic criteria of GBM. Using WHO 2016 criteria, 89 (*n* = 89) cases were ultimately included in the final analysis. Additional information on this clinical series is available separately (Lakis et al., in preparation).

#### 2.12.2 | Immunohistochemistry

Anti-SOX2 (clone 20G5), Thermo Cat# MA1–014, at 1:600 dilution, anti-FOXO3 (FoxO3a (D19A7), Rabbit mAb, Cell Signaling Technology) were obtained commercially and used for immunohistochemistry. Sections were cut at 4-*μ*m thickness, deparaffinized with xylene and rehydrated in ethanol and water. Endogenous peroxidase activity was quenched by incubating slides with Dako Dual Endogenous Enzyme Block. The sections were then incubated with the secondary antibodies in a humidified chamber for 30 min with EnVision Dual Link System-Horseradish Peroxidase (HRP). Antigen-antibody complexes were visualized with peroxidase-based detection systems using diaminobenzidine (DAB) as a substrate.

#### 2.12.3 | Scoring of TMAs

Semiquantitative assessment of the immunohistochemistry results was performed for SOX2 and FOXO3. Only SOX2 nuclear staining was considered positive. The staining intensity was graded as none (0), weak (1+), moderate (2+), or strong (3+) and was multiplied by the percentage of positive cells stained. Scores less than or equal to 100 were considered low SOX2 expression, and scores > 100 were considered high SOX2 expression. FOXO3 nuclear staining was considered positive, and cytoplasmic was not scored. The staining intensity was graded as none (0), weak (1+), moderate (2+), or strong (3+) and was multiplied by the percentage of positive cells. Scores less than or equal to 100 were considered low FOXO3 expression, and scores > 100 were considered high FOXO3 expression.

### 2.13 | Immunofluorescence of SOX2/FOXO3

Frozen tissue specimens: A total of 20 frozen GBM specimens (WHO grade IV) were identified in the Tumor Bank of the Rhode Island Hospital, a collection approved by Institutional Review Board. For immunofluorescence microscopy, specimens were fixed with 4% paraformaldehyde for 24 h at (+4°C) and cryoprotected by incubation in 5% sucrose for 24 h (+4°C). Fixed cryoprotected tissue was stored at −80°C.

#### 2.13.1 | Immunofluorescence staining

Fixed and cryoprotected GBM tissues were sectioned at 5 *μ*m on positive-coated slides and rehydrated for 15 min in phosphate-buffered saline (PBS), pH 7.4. The sections were then post-fixed in 4% paraformaldehyde for 15 min and rinsed in PBS. Non-specific background was blocked by incubation in blocking solution (5% goat serum, 5% donkey serum, 0.3% Triton X-100 in PBS, pH7.4) for 1 h at room temperature. The sections were incubated with anti-SOX2 rat monoclonal antibody (ThermoFisher #14–9811-82, 1:200 dilution) overnight at (+4°C) followed by donkey anti-rat IgG Alexa 488 staining (ThermoFisher #A-21208, 1:1,000 dilution). Labeling of FOXO3 was performed subsequently by using anti-FOXO3 (D19A7) rabbit monoclonal antibody (Cell Signaling Technologies, #12829, 1:200 dilution) 1 h at room temperature. To enhance FOXO3 staining, VectaFluor Excel Amplified AntiRabbit IgG, DyLight 594 Antibody Kit was used according to the manufacturer’s protocol. The slides were mounted with ProLong Glass Antifade Mountant with NucBlue Stain (ThermoFisher #P36981) and cured overnight at room temperature.

#### 2.13.2 | Immunofluorescence quantitation

Dual-labeled immunofluorescence slides were examined with confocal and conventional fluorescence microscopy, using Green Fluorescent Protein (GFP) and Rhodamine filters. For each GBM case, three fields were photographed at 40x and 120x (60 × 2) magnification and the images were used for quantitation. Using Photoshop, the total number of nuclei (identified with NucBlue staining) were counted. Using GFP/rhodamine channels, individual nuclei were identified as negative (blue only), FOXO3 (red), SOX2 (green), or dual-expressing (yellow).

### 2.14 | GSC cultures

Primary GSCs were cultured from human glioma samples as previously described.^57–59^ GSCs were used between passages 12 and 18 for all experiments and cultured as neurospheres in the following conditions: Neurobasal A (Gibco), 2% B27 (Gibco), 2 mM GlutaMAX (Gibco), 2 *μ*g/ml heparin solution (Stem Cell Technologies), 2% Antibiotic-Antimycotic (Gibco), 20 ng/ml Epidermal Growth Factor (EGF) and 20 ng/ml basic Fibroblast Growth Factor (bFGF) (both from Peprotech). For passage, whole neurospheres were dissociated by 2–3 min incubation in Accutase (Life Technologies) coupled with manual trituration, until a single cell suspension was obtained. To induce FOXO3 nuclear localization, cells were incubated in growth media lacking growth factors and/or 20 *μ*M LY294002 (InvivoGen) for 16 h prior to fixation for 10 min in 4% paraformaldehyde.

### 2.15 | Immunocytochemistry

Cells were plated onto fibronectin (Gibco) treated glass coverslips at a density of 50,000 cells/ml. Sixteen hours after plating, cells were fixed with 4% paraformaldehyde for 10 min, followed by 5x washes with PBS. Cells were blocked for 1 h at room temperature with 5% NDS (Jackson ImmunoResearch) in 1X PBS, followed by 5x washes with 1X PBS + 0.05% Tween-20. Cells were then incubated for 2 h in a blocking solution containing primary antibodies: rabbit anti-SOX2 1:200 (EMD Millipore AB5603), mouse anti-HA 1:200 (Roche 12CA5), rabbit anti-FOXO3 1:200 (CST D19A7), rabbit anti-OLIG2 1:500 (EMD Millipore AB9610). After five additional washes with PBS 1X + 0.05% Tween-20 and an additional 10-min incubation in blocking solution, cells were incubated with appropriate secondary antibodies in blocking solution for 1 h at room temperature: 1:500 donkey anti-rabbit 488, donkey anti-mouse 488 (both Jackson ImmunoResearch). Following 5x washes with PBS 1X + 0.05% Tween-20 and 1x wash with PBS 1X, coverslips were mounted onto slides with VECTASHIELD containing DAPI (Vector Laboratories). Imaging was performed on a Zeiss Axiovert 200-M Fluorescence Microscope and ImageJ was used for image analysis.

### 2.16 | Lentiviral overexpression of FOXO3 in GSCs

Lentiviral supernatants were generated in 293T cells as previously described.^30^ Cells were infected with viral supernatants 16 h after plating. Each culture was infected with the transactivator FUW-rtTA at a 1:1 ratio to either FUW-TetO-GFP, FUW-TetO-FOXO3-WT, or FUW-TetO-FOXO3-CA, with the total viral supernatant at a 1:3 ratio to growth media. After 24 h, the virus was removed and replaced with growth media containing 2 *μ*g/ml doxycycline (Sigma). Cells were fixed following 24 h of doxycycline induction. Subsequent immunostaining was performed as described above.

### 2.17 | In vitro proliferation assays

10 *μ*M EdU (Invitrogen) was added to cultures 2 h prior to fixation. EdU detection was performed using the Click-IT Plus EdU Alexa Floura 594 Imaging Kit (Invitrogen) per the manufacturer’s instructions. When performed in tandem with immunocytochemistry, EdU detection was performed prior to blocking. Cells were mounted, imaged, and analyzed as described above.

### 2.18 | Fluorescence-activated cell sorting and NSC analysis

Mice were housed and used for experiments in accordance with a protocol approved by the Brown University Institutional Animal Care and Use Committee. For RNA-seq on mouse NSCs, quiescent and activated NSCs were isolated as described.^56^ Briefly, *Foxo3*^+*/*+^ and *Foxo3*^−/−^ mice carrying the transgenic allele hGFAP-GFP were generated. NSCs were isolated from the adult (7 months) or aged (19–21 months) subventricular zone (SVZ). GFP was used in combination with prominin-1 (CD133) and the EGF receptor to prospectively isolate aNSCs and qNSCs, which can be distinguished by the surface expression of EGFR. Using this scheme, aNSC are prominin-1+; GFP+; EGFR+ and qNSC are prominin-1+; GFP+; EGFR−. Cells were sorted on a BD FACS Aria. The experiment was performed in biological triplicate, generating each library from a single animal, with ~400 cells per library. RNA-seq analysis: Paired-end reads were quality trimmed using Trim galore (http://www.bioinformatics.babraham.ac.uk/projects/trim_galore/) and subsequently aligned to the mouse reference genome, mm10, using HISAT2.^60^ Genes were quantified using HTSeq-Count.^61^ Differential gene expression was determined using DESeq2^62^ and genes that were significantly differentially expressed were defined as False Discovery Rate (FDR)-corrected *p*-value of 0.05. Rank-based analysis was performed using Gene Set Enrichment Analysis.^63^

For mitotracker staining, primary NSCs were isolated from the SVZ as described above and immediately incubated with 500 nM mitotracker deep red (ThermoFisher M22426) for 45 min in Neurobasal A + B27 at 37°C. Cells were washed with PBS and fixed with 4% paraformaldehyde/PBS for 10 min at room temperature. Cells were washed with PBS and permeabilized with 0.2% TritonX in PBS for 10 min at room temperature. Cells were washed with 0.1% TritonX/PBS and incubated with a SOX2 antibody directly conjugated to Alexa488 (EMD Millipore AB5603A4) diluted 1:100 in 10% normal donkey serum, 0.2% TritonX in PBS. Cells were washed with PBS and analyzed on a FACSCalibur flow cytometer and data analyzed using Flowjo software (v10).

### 2.19 | GSC shRNA experiment and RNAseq

GSC-1 cells were infected with shRNA targeting FOXO3 or EV. The amount of virus used for each respective condition was chosen to reproduce similar amounts of GFP signal and percent infection rate. Four days post-infection of shRNA constructs, FOXO3-KD and EV GSCs were collected for RNA-sequencing. An additional FOXO3-KD and EV timepoint was collected 8 days post infection, but only the FOXO3-KD samples were sent for sequencing at this time point. Western blotting was performed to confirm the knockdown of FOXO3 protein at all time points (rabbit anti-FOXO3; CST 75D8; 1:1000). The experiment was performed with three independent samples per condition. Samples were prepped using the RNeasy kit (Qiagen). Transcripts were processed using the new Tuxedo protocol.^64^ Illumina adapters were removed from RNA-seq reads using TrimGalore! (version 0.4.0, Babraham Bioinformatics) and aligned to the human reference genome, hg19, using Hisat2 (version 2.1.0).^60^ Aligned reads were assembled using StringTie (version 1.3.4d), and the expression levels of all genes and transcripts were estimated as previously described.^64,65^ Differential expression of expressed genes was determined using DESeq2, with FDR < 0.05.

### 2.20 | CRISPR/Cas9 ablation of FOXO3 in GSCs and associated functional assays

The lentiCRISPRv2 (one vector system; Addgene plasmid # 52961) was used for CRISPR/Cas9 ablation experiments. CHOPCHOP was used to identify six candidate target sites within the endogenous human FOXO3 locus^66^ and generate independent guide RNAs and a scrambled control sequence. Guides were cloned into the lentiCRISPRv2 vector using published protocols from the Zhang lab.^67,68^ LentiCRISPRv2 constructs were first screened in u87MG cells (ATCC) to identify a guide RNA that results in ablation of FOXO3. Western blotting was performed against FOXO3 to identify a guide that knocked out FOXO3 but not the other related FOXOs (FOXO1 and FOXO4). Lentiviruses were generated as described above and GSC-1 or GSC-2 cells were infected with viral supernatants for 24 h, replaced with basal media, and puromycin selected for 14 days. Following puromycin selection, cells were then expanded for experiments. Cell proliferation was assessed using a 2 h EdU incorporation assay (as described above). ATP production assays were performed using the Luminescent ATP Detection Assay Kit (ab113849; Abcam). GSC-1 FOXO3-knockout or control cells (FOXO3-SC) were plated onto 96 well plates, cultured overnight, and the ATP assay was performed according to the manufacturer’s protocol, and luminescence was quantified using a Cytation 5 cell imaging multi-mode plate reader. ATP levels were normalized for cell number (per 10,000 cells) to correct for potential proliferation differences in control (FOXO3-SC) or FOXO3-ablated GSCs.

## 3 | RESULTS

### 3.1 | Identification of a FOXO3-associated genomic program enriched for quiescence signatures and restricted oxidative metabolism in GBM

**FIGURE 1:**
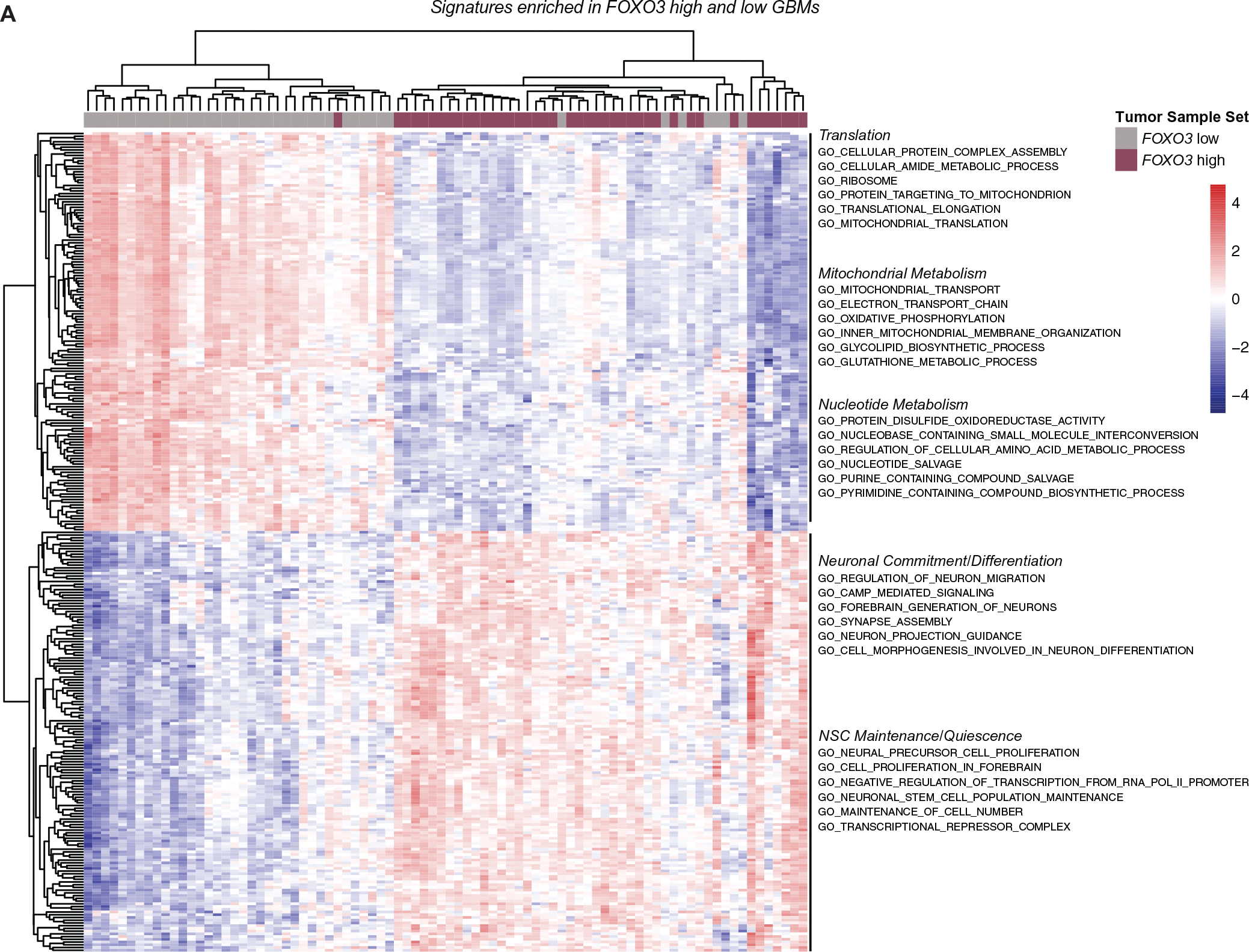
Transcriptome analysis of glioblastoma (GBM) tumors reveals a FOXO3-associated transcriptional program that restrains cell cycle progression and has a distinct mitochondrial metabolism. Single sample gene set enrichment analysis analysis showing the top 300 differentially regulated gene ontology (GO) categories with adjusted *p* < 0.0001 between FOXO3 high (red) and FOXO3 low (grey) GBM tumors

FOXO3 is a well-established regulator of mammalian stem cells and functions to preserve stem cell pools in the brain during aging. To understand FOXO3’s function in GBM, we analyzed publicly available RNA-seq data from 169 human GBM tumor samples from the Cancer Genome Atlas (TCGA).^69^ We first split the GBM tumor samples based on *FOXO3* expression level and compared tumors in the top quartile of *FOXO3* expression (*FOXO3* high) with those in the bottom quartile (*FOXO3* low). To identify the global transcriptional programs and functional pathways that were differentially enriched between the *FOXO3* high and *FOXO3* low tumor samples, we performed ssGSEA.^45^ ssGSEA executes GO enrichment analysis across samples and specifies an enrichment score for each tumor within each GO category. When followed by a differential enrichment analysis, this approach allows the identification of pathways that are coordinately up-or downregulated between two populations of samples. ssGSEA analysis comparing *FOXO3* low and high tumors revealed three biological functions that are clearly different in the two sets of tumors: (1) mitochondrial metabolism and translation, (2) nucleotide metabolism, and (3) stemness functions (neuronal commitment and differentiation and NSC maintenance and quiescence; Figure 1). Enrichment for categories associated with nucleotide and mitochondrial metabolism was observed in tumor samples with low *FOXO3* expression (Figure 1A). These samples displayed strong enrichment for categories associated with mitochondrial oxidative phosphorylation (oxPhos), electron transport chain (ETC), mitochondrial translation, and ribosomal translation activity (Figures 1A and S1A). In contrast to the metabolic signatures of *FOXO3* low tumors, *FOXO3* high tumor samples displayed enrichment for pathways associated with maintenance of stemness and restraint of cell cycle progression (Figure 1A). Closer examination of these categories revealed two distinct functional groups: (1) a signature associated with stem cell quiescence and (2) hallmarks of neuronal differentiation and maturation (Figure 1A). For example, there was a strong enrichment for processes associated with neuronal fate commitment and differentiation, such as axon guidance, synapse maturation, and neuronal migration (Figures 1A and S1B). Moreover, features of the quiescent state such as high fatty acid beta-oxidation, autophagic flux, cell cycle restraint, and transcriptional repression^70^ were upregulated in the *FOXO3* high tumor samples (Figure 1A and S1C). Overall, *FOXO3* high tumors tended to have elevated FOXO3 protein levels as well (Figure S1D), further supporting distinct quiescence-like hallmarks of *FOXO3* high GBMs.

**FIGURE 2:**
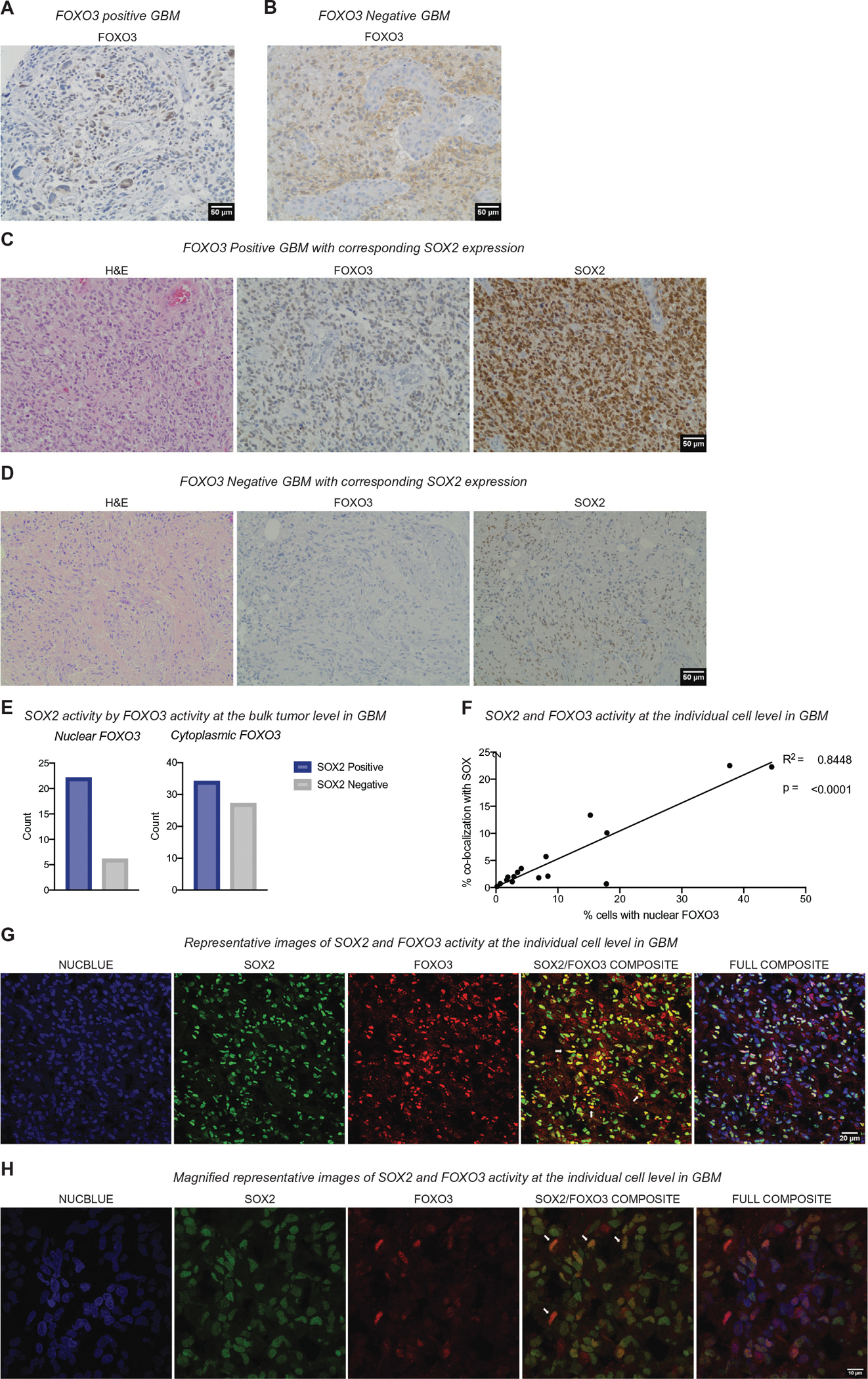
FOXO3 activity predicts SOX2 activity in GBM. (A) Immunohistochemistry of FOXO3 in a GBM tumor sample showing strongly positive (nuclear; active) FOXO3. (Scale bar 50 *μ*m). (B) Immunohistochemistry of FOXO3 in a GBM tumor showing only weak cytoplasmic expression of FOXO3 in the cytoplasm of many tumor cells but not in the tumor cell nuclei or in adjacent endothelial cells. (C) Immunohistochemistry of stemness markers FOXO3 and SOX2, and the corresponding hematoxylin and eosin (H&E) staining in a GBM with high FOXO3 positivity (nuclear; active), and (D) immunohistochemistry of FOXO3 and SOX2 and the corresponding H&E staining in a GBM with low FOXO3 (negative). (E) Quantitation of SOX2 and FOXO3 protein expression by immunohistochemistry identifying FOXO3 activity as nuclear (active; positive) or cytoplasmic (inactive; negative) and strong SOX2 nuclear expression as positive or negative. In tumors with strong FOXO3 expression, SOX2 expression is also usually strongly expressed. (F) Relationship between the percentage of cells with nuclear FOXO3 and the percent of cells showing co-localization of SOX2 and FOXO3 within the same cell as indicated by dual-label immunofluorescence experiments as illustrated in (G). (G) Confocal immunofluorescence images showing FOXO3 (red) and SOX2 (green) co-localization at the individual cell level in GBM tumor samples. In the merged FOXO3/SOX2 images, tumor cells co-expressing FOXO3 and SOX2 are yellow. (Scale bar 20 *μ*m). (H) Higher magnification of confocal immunofluorescence images showing FOXO3 (red) and SOX2 (green), and co-localization (yellow; scale bar 10 *μ*m)

Since we observed that FOXO3 levels correlate with metabolic and stemness signatures in GBM, we investigated FOXO3 expression in GBM tumors and the extent to which it correlates with stemness markers. Using TMAs constructed from human GBM formalin-fixed paraffin-embedded tumor samples (*n* = 89), we used semiquantitative immunohistochemistry to analyze the level of protein expression of SOX2 and FOXO3. We selected SOX2 as a marker of stem-like cells because it has been used as a GSC marker in a number of studies and has been proposed to be a therapeutic target in GBM.^23,71,72^ Many cases exhibited strongly positive FOXO3 staining, where FOXO3 was clearly localized to the nucleus of tumor cells (Figure 2A–C). Because FOXO3 activity is regulated post-translationally through subcellular localization, with active FOXO3 in the nucleus, the *FOXO3* expression analysis included both the intensity of protein expression and its intracellular location. Under conditions that reduce FOXO3 activity (e.g. active growth factor signaling), FOXO3 shuttles into the cytoplasm and can be observed throughout the cell. We, therefore, scored tumors based on the extent to which FOXO3 was located in the nucleus as either strongly nuclear in tumor cells (high activity, Figure 2A) or mostly cytoplasmic in tumor cells, often weakly expressed (low activity, Figure 2B,D). We then used semiquantitative immunohistochemistry to measure SOX2 levels in each sample and scored them as either SOX2 high-expressing (SOX2-positive) or low-expressing (SOX2-negative) GBMs. We observed that most tumors with high FOXO3 expression had high SOX2 protein expression in GBM, while low FOXO3 expression was not related to SOX2 expression (*p* < 0.05, Chi-square; Figure 2E). To determine whether the observed co-expression was within the same cells, we investigated the co-localization of FOXO3 and SOX2 in GBM. Using a second set of GBM tumor samples (*n* = 20, samples frozen at –80°C), we used dual-label immunofluorescence to evaluate the expression of FOXO3 and SOX2 within the same tumor region and cell. We observed a range of percent FOXO3-positive nuclei among samples (0.19%–44.51%), with a median of 5.50% (Figure S1E). We confirmed that *FOXO3* expression often co-localized with SOX2 in the nucleus within the same cells in GBM samples (Figure 2G,H). Within each of these tumors, we counted the number of FOXO3-only expressing cells, the number of SOX2-only expressing cells, and the number of co-expressing cells. Tumors with a high frequency of nuclear FOXO3-expressing cells had a higher degree of nuclear co-localization of FOXO3 and SOX2 (*p* < 0.0001; Figure 2F). These data suggest that GBMs contain populations of stem cells with frequent co-localization of FOXO3 with SOX2 expression and that there was a strong relationship between FOXO3 expression and SOX2 co-expression. In particular, the degree of FOXO3 expression is associated with co-expression of SOX2. Together with the TCGA RNA-seq analysis, these findings indicate that the population of slower cycling stem-like cells may be regulated by a FOXO3-SOX2 common pathway.

**FIGURE 3:**
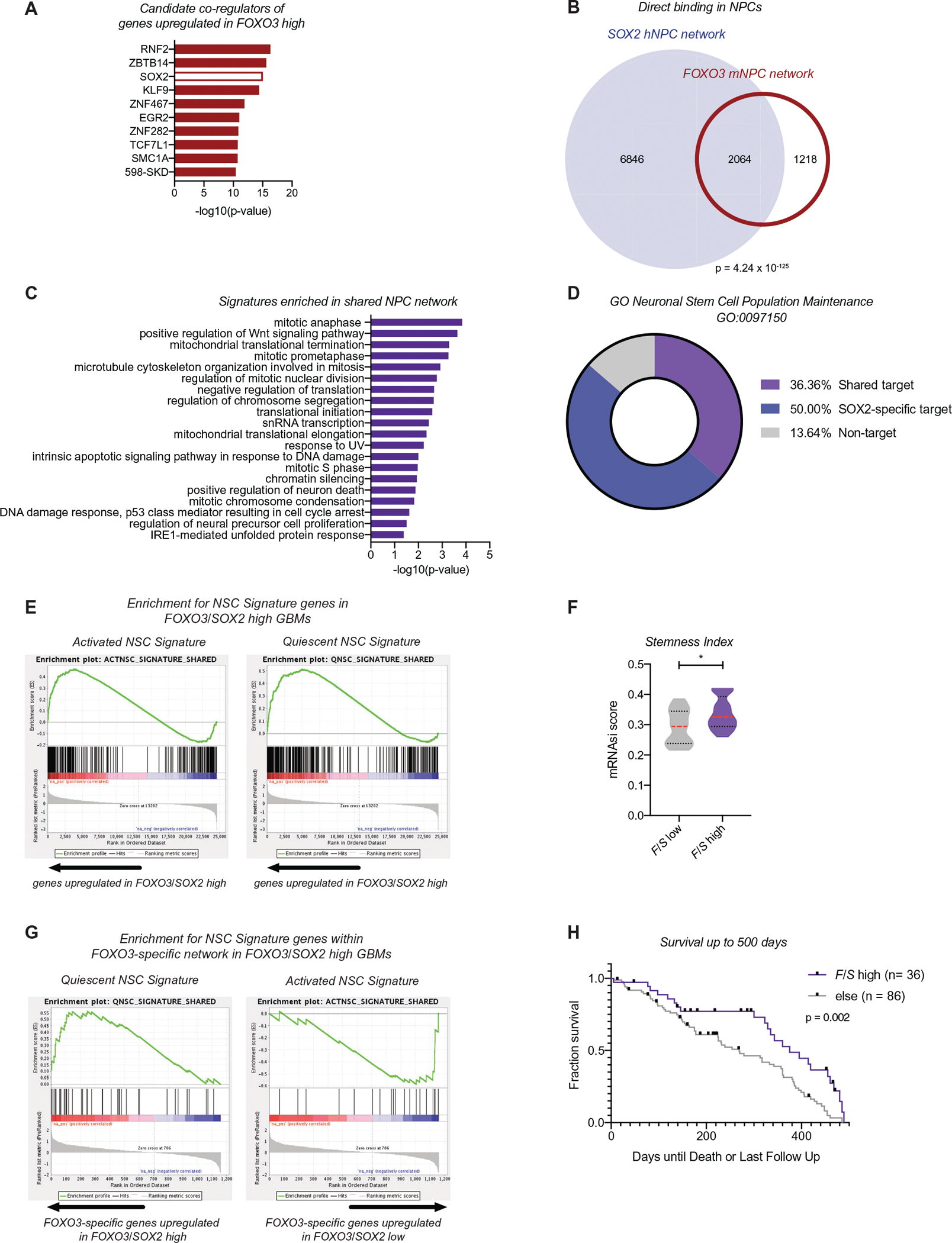
SOX2 and FOXO3 co-regulate a stemness network in neural progenitor cells (NPCs) and GBMs. (A) Landscape in silico deletion analysis analysis of the FOXO3-associated The Cancer Genome Atlas Project (TCGA) GBM network identifies candidate transcriptional co-regulators. The top 10 most significant transcription factors are shown. The *p*-value of the most enriched dataset for each factor is represented. (B) Venn diagram depicting significant overlap between the genes that are direct targets of SOX2 and FOXO3 in NPCs. *p* = 4.24 × 10^−125^, Fisher’s exact test. (C) PANTHER biological process analysis of the 2064 shared direct targets of FOXO3 and SOX2. GO terms displayed based on the hierarchical organization with Bonferonni corrected *p* < 0.05. (D) Enrichment of the shared NPC network, FOXO3-specific network, SOX2-specific network, or non-targets in the neuronal stem cell population maintenance term (GO:0097150). (E) GSEA analysis reveals enrichment for genes involved in NSC quiescence or activation are upregulated in tumors with high *FOXO3/SOX2* expression. (F) Stemness mRNA signature index in *FOXO3/SOX2* high and low tumor samples. Mann–Whitney test at *p* < 0.05, *p = 0.0271. (G) GSEA plot shows an enrichment for quiescence signatures and depletion of activated signatures in samples with high expression of *FOXO3/SOX2* regulated by the FOXO3-specific network. (H) Survival analysis of *FOXO3/SOX2* high TCGA GBMs, compared to all other tumors in the first 500 days. **p = 0.0024. Log-rank (Mantel–Cox) test performed on all survival analyses with *p* < 0.05 considered significant. Censored points shown are days until the last follow-up where days until death was not applicable/available

In parallel to the GBM tumor analysis, we performed an *in silico* analysis of the FOXO3 TCGA network to identify the transcriptional co-regulators that coordinate with FOXO3 to instruct a stemness program. We used epigenetic LISA to identify key transcription factors that bind genes significantly upregulated in the *FOXO3* high GBM tumor samples.^46^ LISA analysis revealed a significant enrichment for members of the kruppel-like zincfinger, Sp1 zincfinger, and SOX families, as well as a member of the polycomb repressive complex (Figure 3A and Table S1). Interestingly, multiple independent datasets identified SOX2 as a candidate co-regulator of the FOXO3-associated network in GBM, including data from human NPCs (Figure 3A and Table S1). Given SOX2’s well-established function in stem cells,^73,74^ we investigated whether SOX2 and FOXO3 share a direct transcriptional network by comparing chromatin occupancy for each factor. To do so, we reprocessed publicly available SOX2 ChIP-seq data in NPCs according to established pipelines in our laboratory and directly compared it to FOXO3 ChIP-seq data we generated previously in NPCs.^30,47^ This approach allowed us to identify and compare direct networks in relatively pure cell populations with stem/progenitor characteristics. We identified 8910 direct SOX2 target genes, of which 2064 (23%) are also FOXO3 targets, revealing a statistically significant overlap between the two networks (*p* = 4.24 × 10^−125^, Fisher’s exact test; Figure 3B and Table S2). This shared network represents 63% of all FOXO3 targets identified in NPCs (Figure 3B and Table S2). PANTHER analysis of the overlapping networks revealed that FOXO3 and SOX2 share direct target genes involved in the regulation of proliferation, the DNA damage response, and translation (Figure 3C). In addition, TCGA GBM samples with high expression of both *FOXO3* and *SOX2* (*FOXO3/SOX2* high) displayed gene signatures associated with stemness, including features of both actively dividing stem cells and those in quiescence (Figure 3D–F). In contrast, GSEA analysis of the FOXO3-specific network (1218 genes in Figure 3B) revealed a quiescence-specific program (Figures 3G and S2A–D), suggesting that within the genomic FOXO3/SOX2 network, FOXO3 binding is associated with the slowly cycling state. Further, we observed that high *FOXO3/SOX2* expression in GBM is associated with improved survival over the first 500 days (*p* = 0.002; Figures 3H and S2E,F). Together, these data suggest that co-localization of these two factors may identify quiescent and/or slowly cycling GSCs within a tumor population. The better survival of individuals with high quiescent content (*FOXO3/SOX2*) GBMs early in their course is suggestive of increased dormancy in these tumors.

### 3.2 | Overexpression of FOXO3 promotes cell cycle arrest in GSCs

**FIGURE 4:**
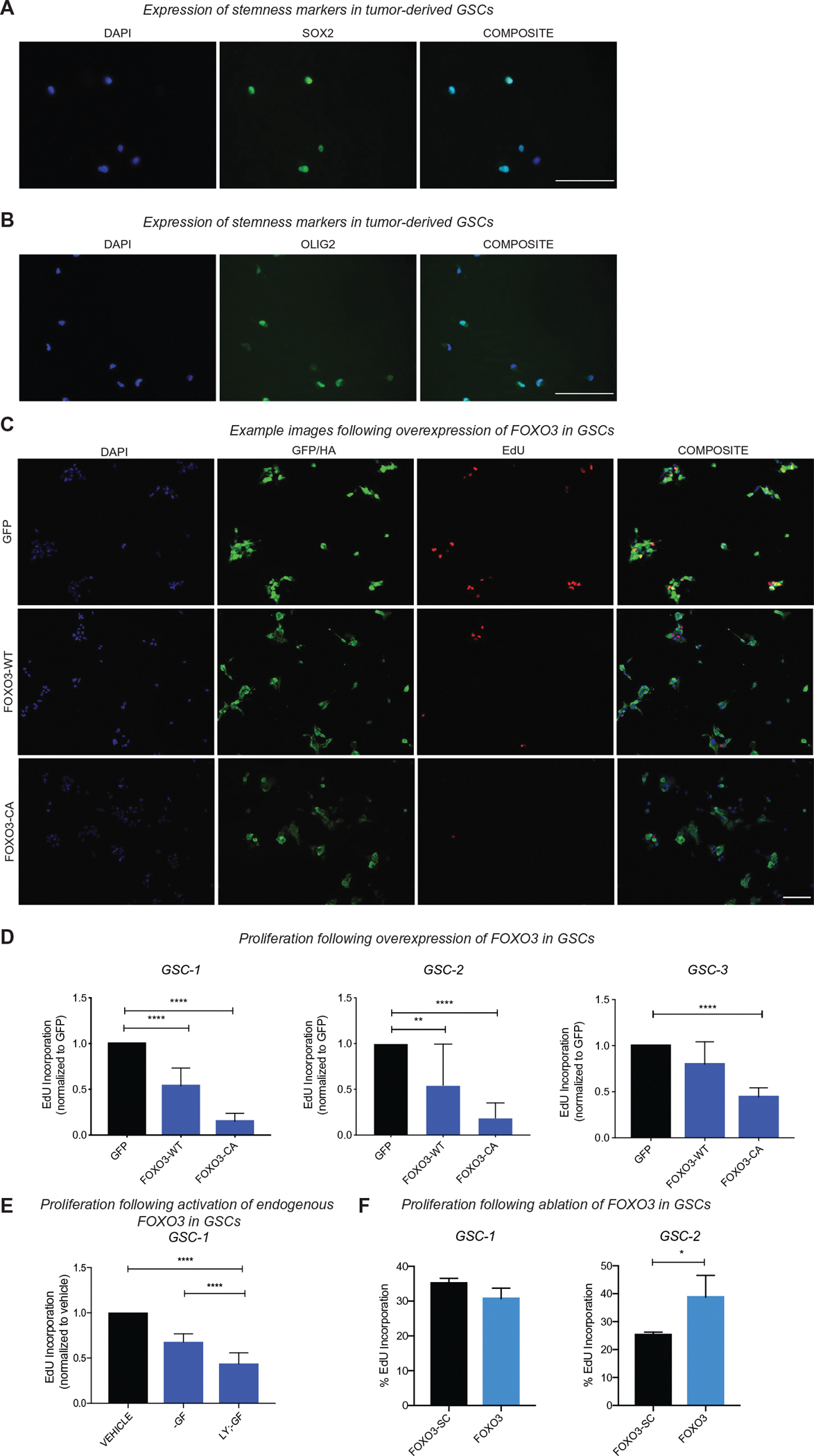
Overexpression and activation of endogenous FOXO3 reduces proliferation in primary human glioma stem cells (GSCs) in vitro. (A) Immunocytochemistry of stem/progenitor markers SOX2 (green) DAPI (blue) and (B) OLIG2 (green) and DAPI (blue) in the primary human GSC culture GSC-1. (Scale bar 100 *μ*m). (C-D) Quantification of cell proliferation (EdU+/total DAPI) upon overexpression of GFP, wild-type FOXO3 (FOXO3-WT) or constitutively active FOXO3 (FOXO3-CA) in three independent GSC populations. One-way Analysis of Variance (ANOVA) with Dunnett’s multiple comparison test at *p* < 0.05, GSC-1 ****p < 0.0001; GSC-2 ****p < 0.0001, **p = 0.0035; GSC-3 ****p < 0.0001. GSC-3–two biological replicates. (Scale bar 100 *μ*m) (E) Quantification of cell proliferation (EdU+/total DAPI) following activation (induced nuclear localization) of endogenous FOXO3 in GSC-1 through growth factor deprivation (−GF) and/or treatment with the PI3K-inhibitor LY294002 for 16 h. One-way ANOVA with Dunnett’s multiple comparison test at *p* < 0.05, ****p < 0.0001. All above experiments were performed in three biological replicates per condition. (F) Quantification of cell proliferation (EdU+/total DAPI) following ablation of FOXO3 (FOXO3) or control (FOXO3-SC) in GSC-1 or GSC-2. Student’s *t*-test, three replicates per cell line. *p* < 0.05

To functionally confirm our bioinformatics analysis, we obtained three independent GSC lines derived from GBM tumors. We first confirmed that these cell populations expressed the stem/progenitor marker SOX2, and have high levels of OLIG2, another established GSC marker^14^ (Figure 4A,B). To examine the effect of FOXO3 activation in GSCs, we used a doxycycline-inducible lentiviral system to overexpress GFP, wild-type FOXO3, or a constitutively active (CA) form of FOXO3 (FOXO3-CA).^30^ The CA form of FOXO3 includes three point mutations in the AKT phosphorylation sites (Thr32, Ser253, and Ser315 are mutated to alanine), which renders it resistant to inhibition by AKT and causes constitutive nuclear localization.^75^ After 22 h, we quantified GSC proliferation by EdU incorporation in each condition. We found that overexpression of wild-type FOXO3 significantly reduced proliferation in two out of three independent GSC lines when compared to overexpression of GFP (GSC-1 and GSC-2; Figure 4C,D). There was a further reduction of proliferation in all three GSC lines upon overexpression of FOXO3-CA (Figure 4C,D).

To confirm our findings, we used pharmacological manipulation in conjunction with growth factor deprivation to induce the activity of endogenous FOXO3. Growth factor deprivation and/or treatment with the PI3K inhibitor LY294002 induce a re-localization of FOXO3 into the nucleus.^30^ Growth factor deprivation decreased proliferation when compared to control conditions and PI3K inhibition caused an even greater decline (Figure 4E). Thus, blocking PI3K/AKT signaling, which correlates with induction of endogenous FOXO3 activity, reduces proliferation in GSCs. Altogether, these data suggest that in populations of GSCs highly expressing SOX2, activation of FOXO3 restricts cell cycle progression.

Since the reduction in EdU incorporation observed in response to FOXO3 activation may be due to cell cycle exit, differentiation, or apoptosis, we further investigated the possibility that FOXO3 regulates proliferation using an ablation approach. We ablated FOXO3 in GSCs using the lentiv2 CRISPR/Cas9 gene editing system. We screened for guide RNAs that effectively target FOXO3 by western blot (Figure S3A). We then measured EdU incorporation in two independent GSC lines, compared to controls. FOXO3-ablated cells showed an increase in cell proliferation in one of two independent GSC lines tested (*p* < 0.05; Figure 4F). This finding is consistent with the notion that FOXO3 can function to restrain proliferation, at least transiently, in some GSC cell lines (GSC-2 in this study). However, this result also suggests that FOXO3 activity is not a major mechanism of grown inhibition in some patient-derived GSCs (e.g., GSC-1).

### 3.3 | Loss of FOXO3 induces a rewiring of transcriptional networks associated with a cell fate shift in GSC-1

**FIGURE 5:**
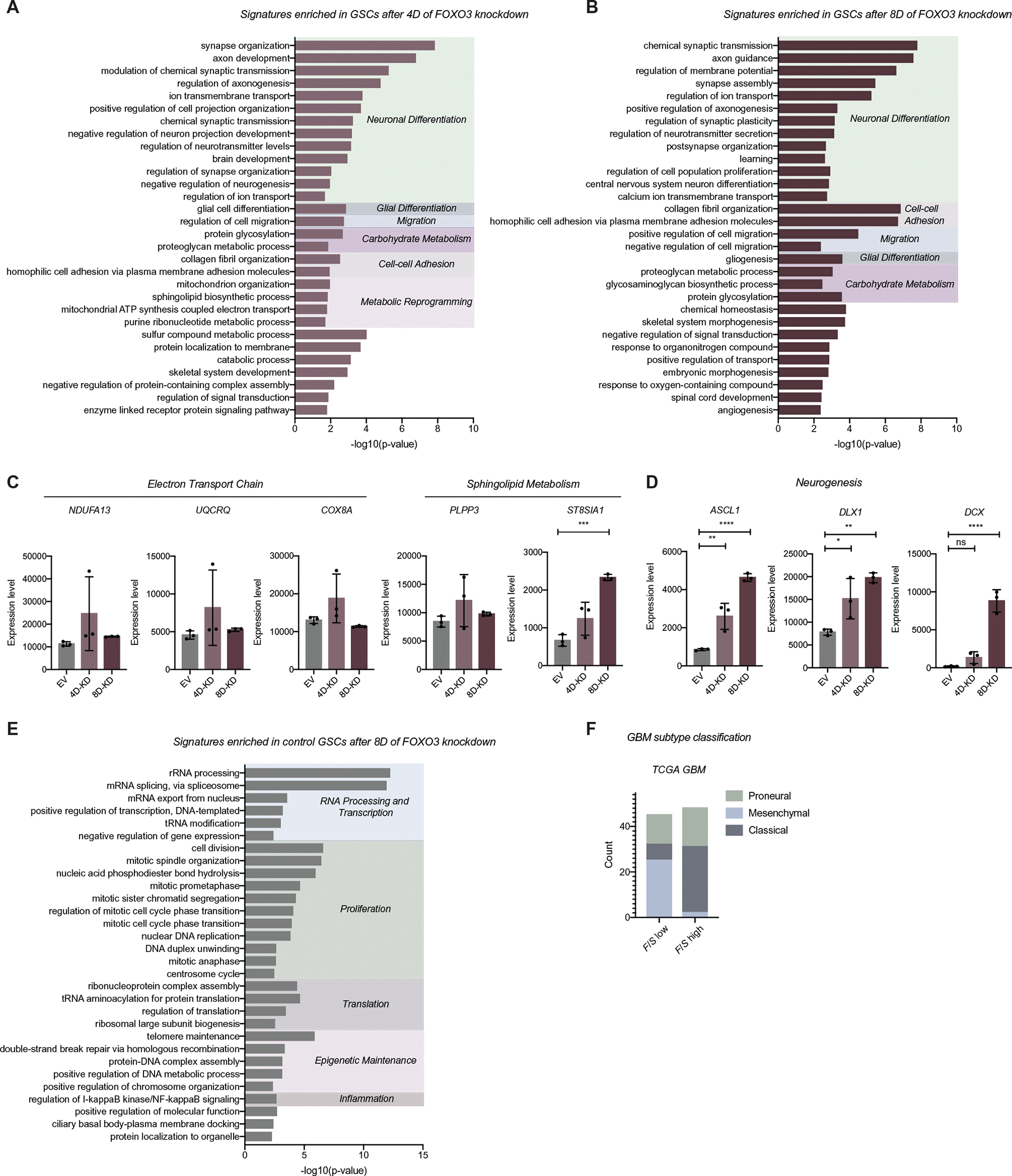
FOXO3 restrains metabolic activation and loss of stemness in GSCs. (A-B) PANTHER biological process analysis of the differentially expressed genes upregulated in FOXO3-KD GSCs after 4 (A) or 8 (B) days of FOXO3 knockdown in human GSCs. (C-D) The expression of example genes associated with metabolic reprogramming (C) and neurogenesis (D) that are altered upon ablation of FOXO3 in GSCs. Expression level indicates gene count value. One-way ANOVA with Dunnett’s multiple comparison test. *p < 0.05, **p < 0.01, ***p < 0.001, ****p < 0.0001. (E) PANTHER biological process analysis of the differentially expressed genes upregulated in control samples (EV) after 8 days of FOXO3 knockdown in human GSCs. For PANTHER analysis above, all genes with adjusted *p*-value < 0.05 were considered significant. The top 30 most significantly enriched GO terms displayed based on the hierarchical organization with Bonferonni corrected *p* < 0.05. Terms were then grouped by function. (F) GBM subtype classification count for TCGA GBM samples with high *FOXO3/SOX2* expression (*F/S high*) or low *FOXO3/SOX2* expression (*F/S low*)

Since FOXO3 ablation in GSC-1 did not affect cell proliferation, we investigated the function of FOXO3 in this line using transcriptional profiling. We performed knockdown experiments using shRNAs against FOXO3 in GSC-1, followed by RNA-seq. Use of shRNAs for this experiment allowed us to perform a precise time course of FOXO3 ablation. We compared control GSCs with GSCs treated for 4 or 8 days with FOXO3 shRNA and performed the experiment in triplicate with independent shRNA knockdowns (FOXO3-KD; Figure S3B–D). PANTHER analysis revealed that genes associated with metabolic reprogramming, cell-cell adhesion, and neuronal differentiation processes were upregulated following 4 days of FOXO3-KD when compared with the control (Figure 5A–C). After 8 days of FOXO3-KD, in addition to metabolic reprogramming and cell-cell adhesion signatures, genes associated with neuronal maturation, circuit integration, migration, and glial differentiation become highly upregulated (Figure 5B–D). These data indicate that loss of FOXO3 in the GSC-1 line initially promotes metabolic activation associated with a less quiescent state, followed by a loss of stemness and neuronal differentiation. Consistent with the loss of stemness, we observed a rewiring of key regulators of stem cells and the SOX2 transcriptional network after 8 days of FOXO3 knockdown, where a significant number of differentially expressed genes were markers of stemness or SOX2 targets (45% of differentially expressed genes are SOX2 targets, *p* = 4.373483 × 10^−35^ Fisher’s exact test; Figure S4A,B). In agreement with a shift away from stemness networks, we found that direct targets of OLIG2^48^ were also differentially regulated after FOXO3 knockdown (Figure S4C). We also note a shift in the expression of genes associated with markers of cell identity, proliferation, cell cycle progression, and self-renewal after 8 days of FOXO3 ablation (e.g., *MCM2* and *JAG1*; Figure S4D). Interestingly, we observed increased levels of markers of differentiating neurons (*DCX*), astrocytes (*GFAP and S100β*), and oligodendrocytes (*MBP*), suggesting that the loss of stemness may result in a heterogeneous population of differentiating cells (Figure S4D). In contrast, processes that were over-represented in GSCs with intact FOXO3 (controls) include translation, RNA processing, and epigenetic maintenance (chromatin modifications, and DNA damage responses; Figure 5E). We further confirmed these findings in GBM tumor samples that were enriched for markers of stemness in the Ivy GAP.^43^ Consistent with our observations, *FOXO3* levels in the Ivy GAP GSCs were associated with cell cycle arrest (high *FOXO3*) and mitochondrial oxPhos and nucleotide metabolism (low *FOXO3*; Figure S5A). Together, this analysis reveals that FOXO3 restrains transcriptional signatures of metabolic activity and differentiation in the GSC-1 line and promotes an expression program that maintains epigenetic stability. Moreover, similar to our analysis of the TCGA dataset, low FOXO3 expression is associated with a specific mitochondrial gene expression profile. These transcriptional programs appear to impact GBM characteristics, as GBMs in the TCGA database with low *FOXO3* levels were frequently characterized as mesenchymal tumors that tend to be more migratory (Figure 5F), and those with high *FOXO3* levels were more often associated with the classical GBM transcriptional profile. Collectively, these data suggest that ablation of FOXO3 in GSCs is associated with loss of stem cell features, but phenotypically, some GSCs increase growth as a result (GSC-2), whereas others exhibit a brief metabolic reprogramming followed by transcriptional rewiring to a more differentiated state (GSC-1).

### 3.4 | FOXO3 shares a transcriptional program associated with metabolic stability in aging and cancer stem cells

**FIGURE 6:**
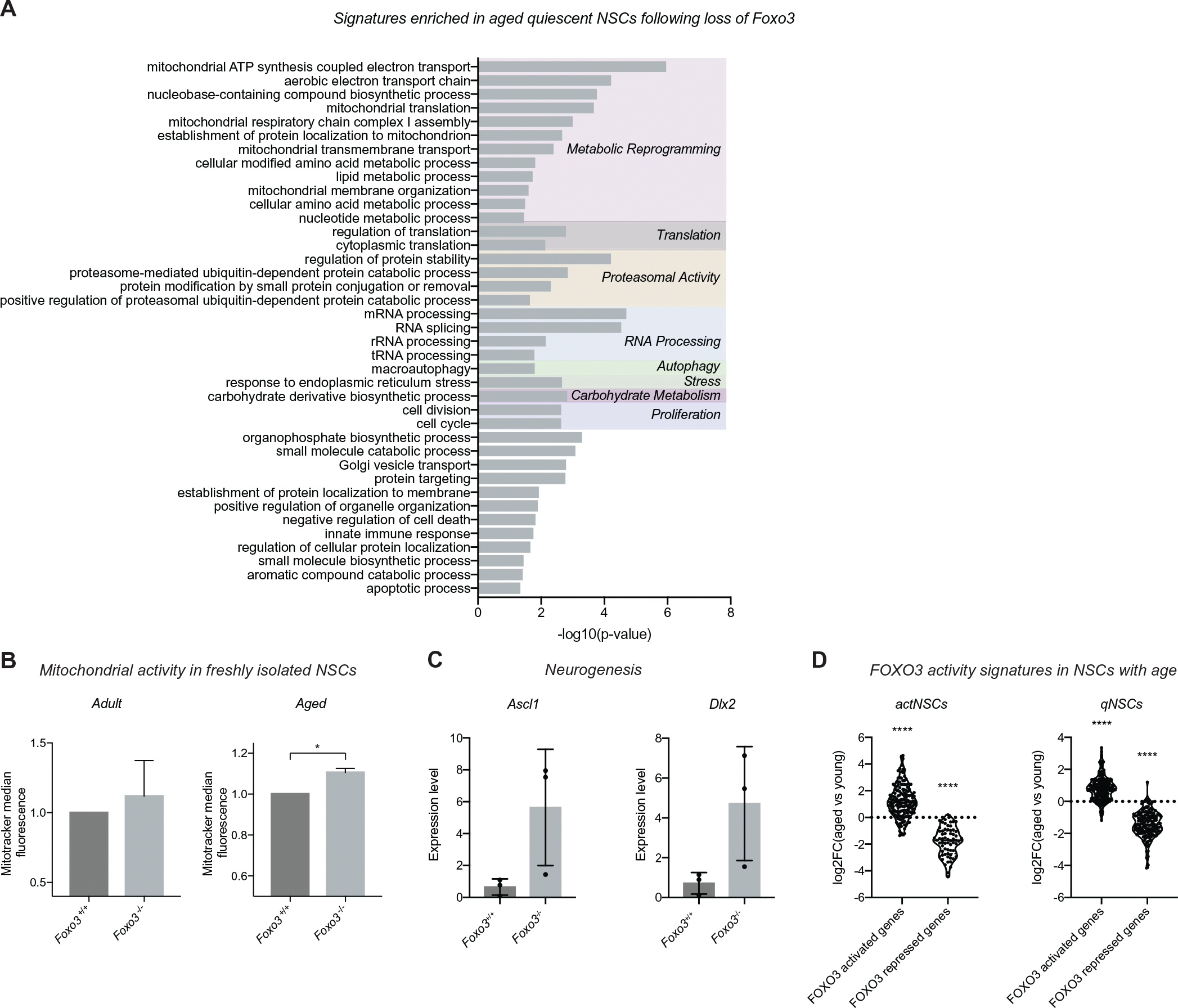
FOXO3 inhibits metabolic drift in endogenous neural stem cells (NSCs) during aging. (A) PANTHER biological process analysis of the differentially expressed genes upregulated in *Foxo3*^−/−^ quiescent NSCs from aged mice. All genes with log2(fold change value) (LFC) of > 0.8 were considered. GO terms displayed based on the hierarchical organization with Bonferonni corrected *p* < 0.05. Terms were then grouped by function. (B) NSCs isolated from aged *Foxo3*^−/−^ mice have increased mitochondrial content (mitotracker fluorescence) compared to wild-type controls. No difference was observed between wild-type and null adult animals. *n* = 6 animals per group; Student’s *t*-test; *p < 0.05. (C) The expression of example genes associated with neurogenesis that are altered in *Foxo3*^−/−^ quiescent NSCs from aged mice. Expression level indicates FPKM value. (D) Age-associated changes in FOXO3 signature genes in activated or quiescent NSCs. Kruskal–Wallis with Dunn’s multiple comparison test where each respective gene set was compared to all non-FOXO3 regulated genes. ****p < 0.0001

Since FOXO3 is a known regulator of healthy aging and stem cell function, our findings raise the question of whether it regulates a shared transcriptional network in GSCs and endogenous NSCs. Further, our observation that FOXO3’s transcriptional network in GSCs includes signatures of metabolic and epigenetic regulation suggests that the GSC network may include features of aging that are associated with the age-associated onset of GBM. Thus, we expanded our study to examine the transcriptional networks regulated by FOXO3 in aged stem cells in vivo. To do so, we freshly isolated quiescent and activated endogenous NSCs from adult (7 months) and aged (19–21 months) *Foxo3*^+*/*+^ or *Foxo3*^−/−^ mice and performed RNA-sequencing (*n* = 3 replicates per group). Similar to our findings in GSCs, genes associated with cellular respiration, mitochondrial oxPhos, and translation were upregulated in *Foxo3*^−/−^ aged quiescent NSCs when compared to their wild-type counterparts (Figure 6A). Interestingly, we did not observe similar metabolic reprogramming in the young quiescent NSCs or either activated population, suggesting that in the absence of FOXO3, quiescent stem cells gradually lose metabolic features of quiescence during the aging process (Figure S6A–C). Consistent with this finding, freshly isolated NSCs from *Foxo3*^−/−^ aged (20 months), but not younger adult (3 months) mice have increased mitochondrial activity, compared to wild-type controls (Figure 6B), as well as increased expression of markers of activated NSCs (Figure 6C).

To begin to understand the similarity between FOXO3’s functions in aging and cancer, we generated FOXO3 transcriptional signatures of quiescent and activated NSCs from the RNA-seq dataset for further analysis. Since we observed the greatest differences in gene expression in the aged NSCs, we defined the FOXO3 signatures as the most differentially expressed (top 200) genes between wild-type and *Foxo3*^−/−^ cells within the aged activated or quiescent populations. First, we observed that the FOXO3 signature genes were altered in expression with age in each respective cell type (Figure 6D), indicating that the FOXO3 transcriptional program is globally affected in aging NSCs. Specifically, we found that FOXO-activated genes were generally increased in expression with age, whereas FOXO3-repressed genes were overall reduced in expression (Figure 6D). Thus, in normal physiological aging, the FOXO3 transcriptional networks are further reinforced in NSCs.

To understand the extent to which the FOXO3 NSC signatures correlate with the GBM transcriptional networks we identified, we calculated an ssGSEA score for the FOXO3-activated and -repressed NSC signatures across the 169 TCGA tumor samples used in our initial analysis (Figure 1). We found a significant correlation between FOXO3 levels in GBM and the NSC signatures, where tumors with low FOXO3 activity (derepression of the FOXO3-repressed signature) also had low FOXO3 expression levels (*p* < 0.0001; Figure S6D). Moreover, the quiescent NSC FOXO3 transcriptional signature predicts similar tumor features as FOXO3 expression in GBM in the TCGA database (Figure S6E). Together, these data show that a FOXO3 signature derived from endogenous NSCs can reliably predict FOXO3 levels and activity in GBM tumors.

**FIGURE 7:**
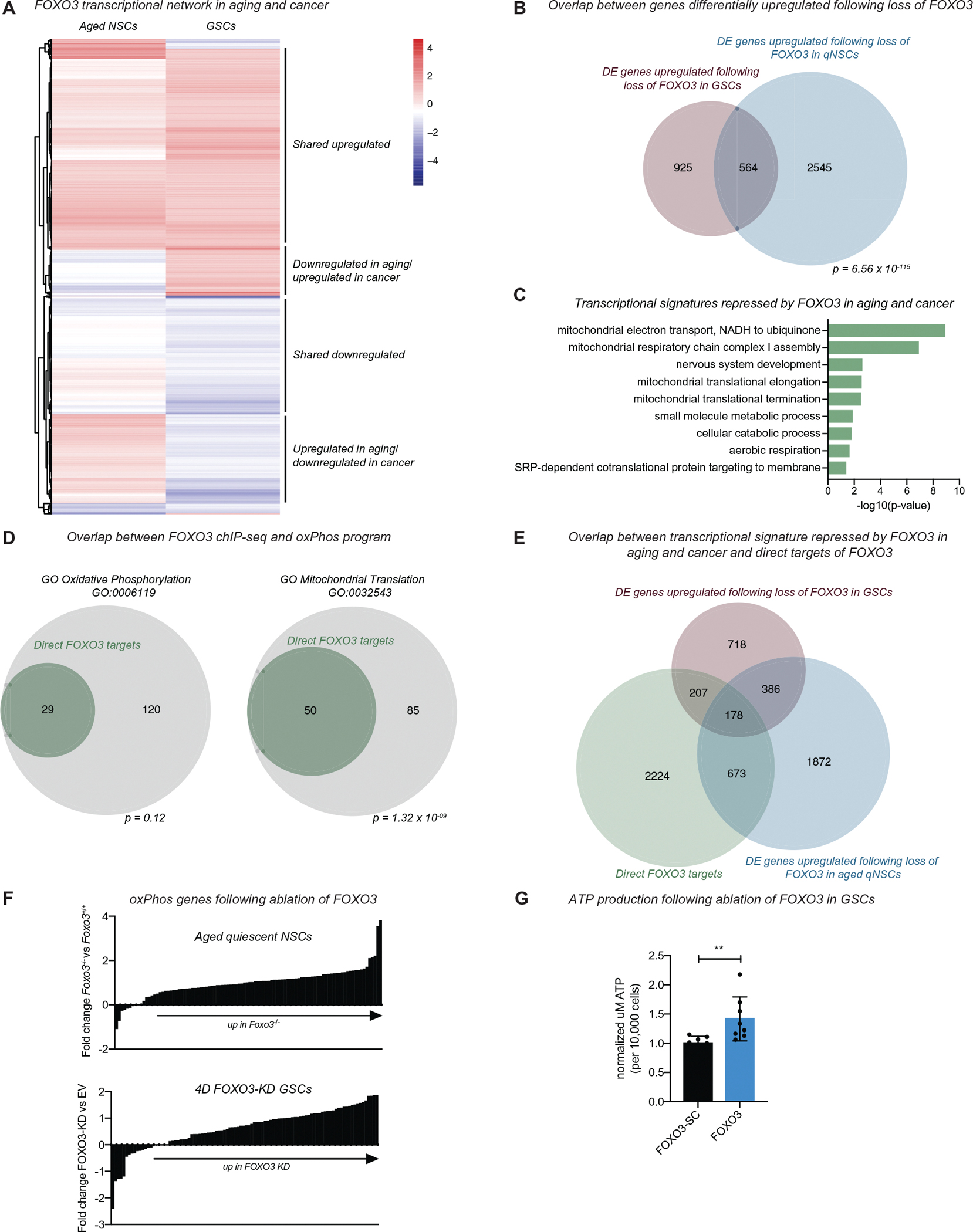
FOXO3 functions as a common node in cancer and aging stem cells. (A) Heatmap depicting genes differentially upregulated in GSCs following 4 days of FOXO3 knockdown (GSCs) and their respective fold change value following the loss of *Foxo3* in aged quiescent NSCs (aged NSCs). (B) Overlap between genes differentially upregulated following loss of FOXO3 in GSCs and aged NSCs. p = 6.56 × 10^−115^ Fisher’s exact test. (C) PANTHER biological process analysis of the 564 genes that are differentially upregulated following 4D of FOXO3-KD in GSCs and loss of *Foxo3* in aged quiescent NSCs in (B). GO terms displayed based on the hierarchical organization with Bonferonni corrected *p* < 0.05. (D) Overlap of FOXO3 ChIP-seq data from endogenous NSCs genes involved in mitochondrial oxidative phosphorylation (oxPhos; left) and mitochondrial translation (right). *p* = 0.12 and *p* = 1.32 × 10^−9^ Fisher’s exact test. (E) Overlap of FOXO3 ChIP-seq data from endogenous NSCs and differentially expressed genes common to aging and cancer. For analysis above, genes with a LFC > 0.8 were considered differentially expressed and upregulated in GSCs or aged NSCs. (F) LFCs in expression of oxPhos genes in aged quiescent NSCs (top) and human GSCs (bottom), comparing FOXO3 ablated cells to cells with intact FOXO3. (G) ATP production assay in GSC-1 following ablation of FOXO3 (FOXO3) or control (FOXO3-SC) GSCs, two biological replicates with four technical replicates per condition. Mann–Whitney test, **p < 0.01

We next compared FOXO3-regulated expression programs in aging and cancer transcriptome-wide. We observed the strongest correlation between genes that are deregulated upon loss of FOXO3 in GSCs and aged quiescent NSCs (Figures 7A and S7A–D,S8) and a statistically significant overlap between these conditions (*p* = 6.56 × 10^−115^ Fisher’s exact test; Figures7B and S9A–C; Table S3). Moreover, we also find subsets of genes that are deregulated exclusively in either GSCs or aged NSCs, thereby representing cancer stem cell-specific or agingspecific FOXO3 networks (Figures 7A and S9D–F). Functionally, genes upregulated in FOXO3-ablated aged quiescent NSCs and GSCs (repressed by FOXO3; 564 genes) were associated with mitochondrial oxPhos, the ETC (mitochondrial respiratory chain complex I assembly), and mitochondrial translation (Figure 7B,C), suggesting stem cell quiescence, both in aging and in gliomas, is associated with a particular mitochondrial state. Since FOXO3 is mostly a transcriptional activator, but the metabolic signatures were significantly upregulated in both datasets in the absence of FOXO3, we then tested whether FOXO3 was directly repressing mitochondrial oxPhos gene expression. We compared the expression datasets to our previously generated ChIP-seq data for FOXO3 and did not find evidence for a network-wide direct repression of oxPhos genes by FOXO3 (*p* = 0.12, Fisher’s exact test; Figure 7D–E). We do note, however, that there is a significant enrichment of binding at the genes involved in the regulation of mitochondrial translation.

Together, these data show that *Foxo3* regulates a transcriptional program involved in metabolic stability of the quiescent NSC pool throughout aging, such that loss of *Foxo3* results in an accumulation of mitochondria and transcriptomic signatures of metabolic reprogramming in old mice. Since a similar transcriptional shift in oxPhos genes was also observed in human GSCs after 4 days of FOXO3 ablation (Figure 7F), we tested whether FOXO3 functionally modulates metabolic states of GSCs. We performed an ATP production assay in GSCs (GSC-1) after ablation of FOXO3 and observed that ATP production increased in GSCs after FOXO3 ablation (*p* < 0.01; Figure 7G). Collectively, our data indicate that a mitochondrial oxPhos gene program is indirectly repressed by FOXO3 in aging and cancer, suggesting that FOXO3 functions upstream in a transcriptional hierarchy that is associated with setting metabolic states in healthy and transformed stem cells.

## 4 | DISCUSSION

Aging is a major risk factor for many cancers^76^ including GBM. During aging, changes to the microenvironment as well as intrinsic alterations to epigenetic and metabolic states increase the propensity for tumor initiation. Tumor initiating cells share many features with healthy somatic stem cells, and GBM can arise through the transformation of endogenous NSCs. Here, we identify FOXO3 as a shared transcriptional node in aging NSCs and GBM.

FOXO3 is a well-established regulator of stem cell pools and aging. Our findings indicate that FOXO3 restrains a transcriptional program that is enriched for genes involved in metabolism and stemness. In healthy adult NSCs, exit from quiescence is associated with a shift from glycolytic to mitochondrial oxPhos transcriptional programs, as well as increased mitochondrial content and ATP production.^55,56,77,78^ GSCs are similarly regulated and can transition between rapidly dividing and slow cycling states.^79^ We found that elevated *FOXO3* expression correlates with transcriptional programs that support a less anabolic state in whole GBMs and GSCs. Functional ablation of FOXO3 rapidly rewires these metabolic transcriptional networks in GSCs. Interestingly, a recent study identified a GBM subtype based on an oxPhos metabolic phenotype that shows selective vulnerability to mitochondrial inhibitors.^42^ Moreover, aging is also associated with changes in mitochondrial function.^80^ Our data suggest a role for FOXO3 in restraining this mitochondrial-associated phenotype, particularly in the context of aging. It remains to be determined whether the link between FOXO3 and oxPhos gene programs is direct in nature since FOXO3 mostly functions as a transcriptional activator and high *FOXO3* levels correlated with low oxPhos expression programs. Instead, transcriptional regulation of metabolic genes may be achieved indirectly, through processes such as protein quality control, which are directly regulated by FOXO3 in healthy NSCs^81,82^ and enriched in the FOXO3 network in GBM. Nevertheless, FOXO3 appears to function as a crucial transcriptional node to safeguard stem cells from metabolic activation over time. These findings raise the possibility that destabilization of the FOXO3 network may predispose NSCs to transformation to tumor-initiating cells. This may have therapeutic implications given the recent links between mitochondrial function, cancer stem cell growth, and therapeutic resistance,^83^ and the recent identification of a mitochondrial-pathway subtype in GBM.^42^

Our results also associate low FOXO3 activity with transcriptional signatures of neuronal differentiation in some GSC lines and a cell fate shift away from quiescence in GSCs. In contrast, signatures of epigenetic maintenance were higher in the GSCs with intact FOXO3 activity, suggesting that FOXO3 maintains epigenetic stability in these cells. Maintenance of epigenetic states is essential for healthy stem cell function and epigenetic dysregulation is a common feature of cancer.^84^ Chromatin changes have been linked to GBM initiation and progression, and GSCs can undergo epigenetic rewiring to evade therapies.^85–88^ Interestingly, GSCs harbor epigenetic profiles that are distinct from other brain tumors and can adopt transcription factor programs similar to endogenous NSCs.^89^ Much of the transcriptional circuitry previously identified involves developmental regulators that support neurogenic fate. In contrast, our work implicates FOXO3, an age-associated transcriptional regulator, as a stabilizer of these transcriptional programs, which may function to preserve genomic stability in the context of damage or stress.

In summary, we have uncovered a novel link between age-associated transcriptional programs in stem cells and GBM-initiating cells. This work identifies FOXO3, an integral regulator of stem cell homeostasis during aging, as an intrinsic regulator of GSCs through a shared transcriptional network. The transcriptional programs identified here provide new insight into the mechanisms supporting tumor growth in aged individuals, and uncover the need to better understand the connection between aging and the potency of tumor-initiating cells.

## Supplementary Material

SuppFig-3

SuppFig-2

SuppFig-4

SuppFig-5

SuppFig-6

SuppFig-7

SuppFig-8

SuppFIg-9

SuppTable-1

SuppTable-2

SuppTable-3

SuppFig-1

## Data Availability

All RNA-seq data included in this manuscript will be made publicly available in GEO at the time of publication.
