# Supplementary figures and images for: FOXO3 regulates a common genomic program in aging and glioblastoma stem cells
Source: Aging Cancer. Author manuscript; Available in PMC 2022 Oct 26. (PMC9601604; doi:10.1002/aac2.12043)

Figure S3

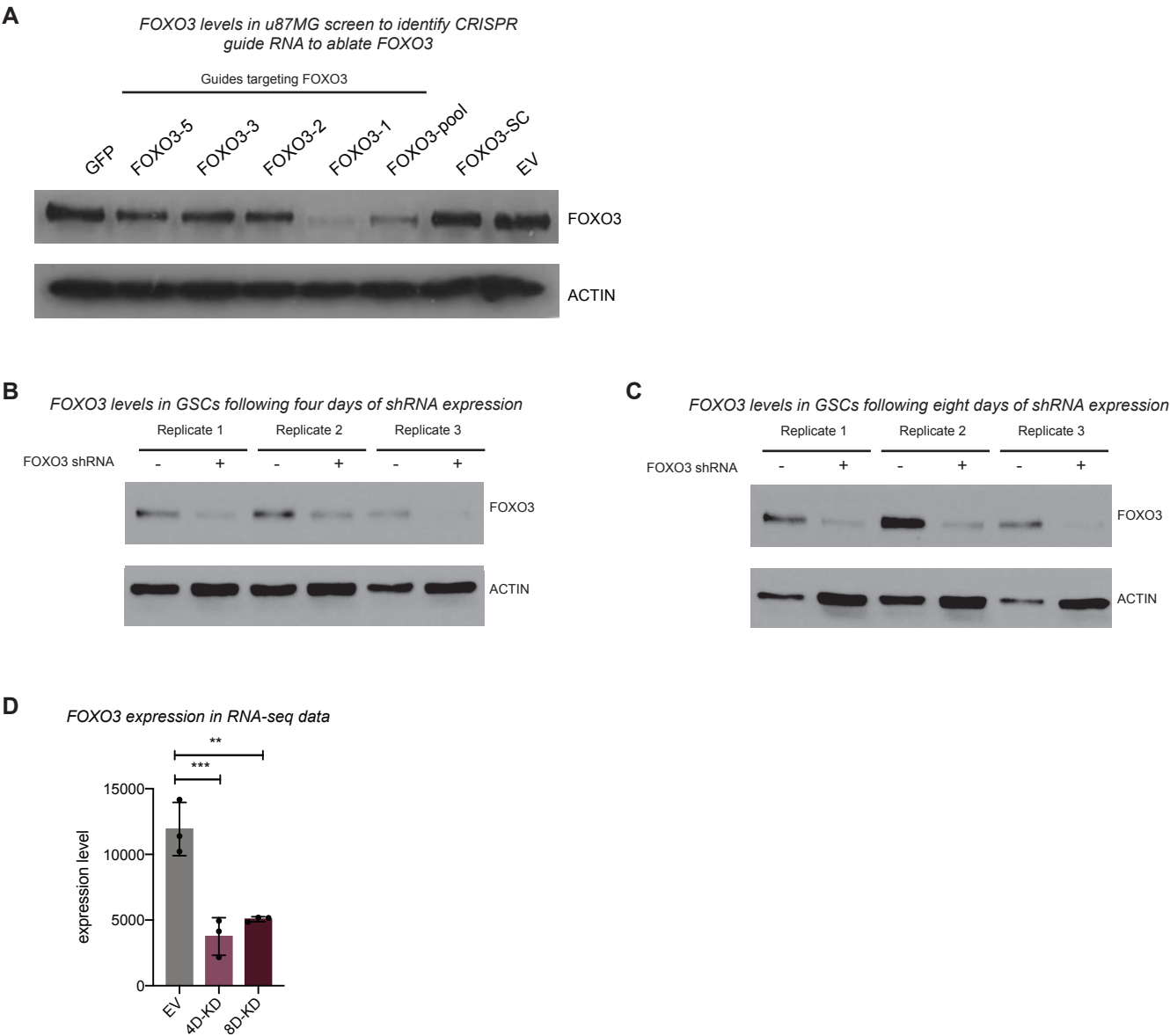

Supplement: SuppFig-3 [file NIHMS1841143-supplement-SuppFig-3.pdf]

Figure S2

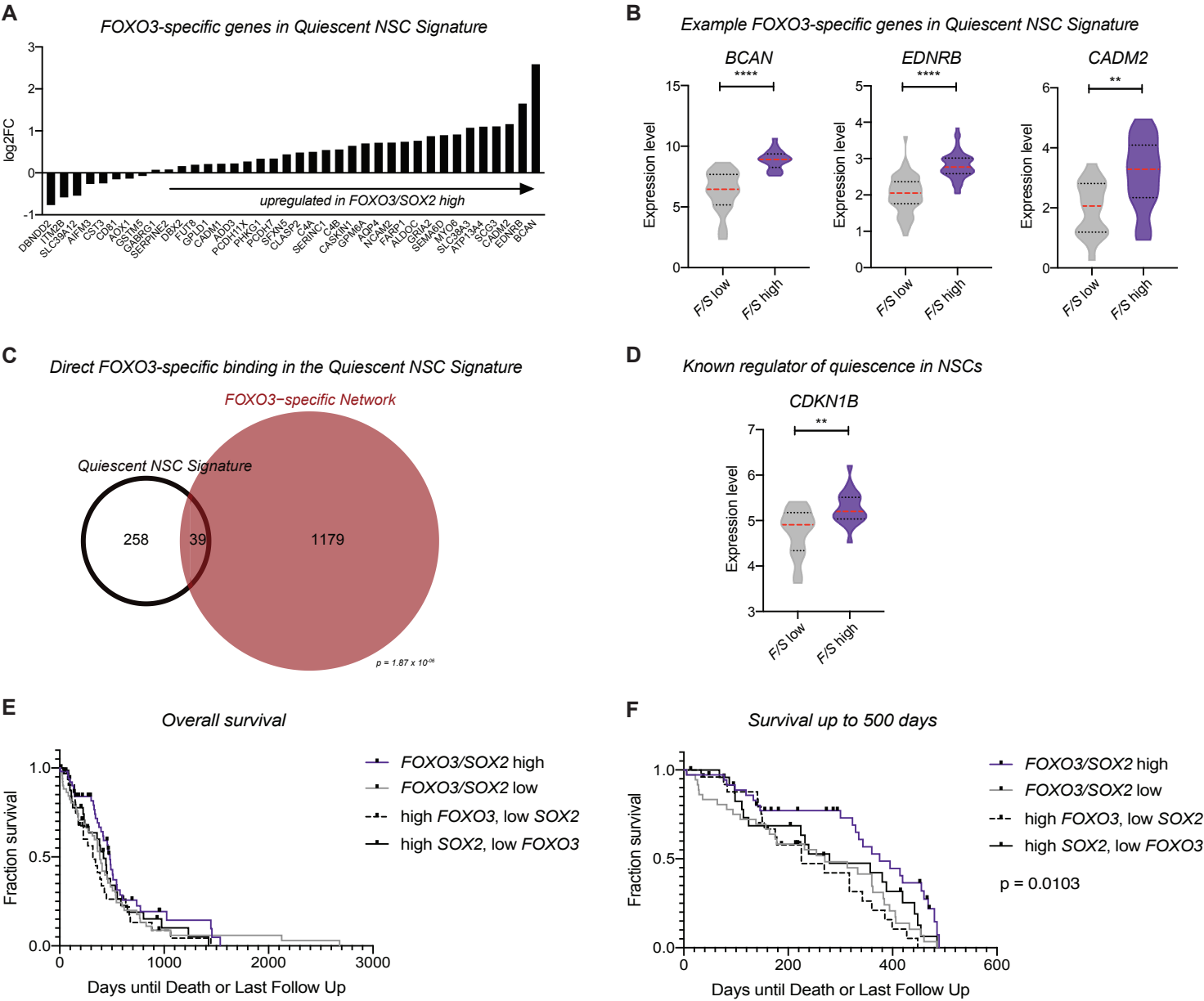

Supplement: SuppFig-2 [file NIHMS1841143-supplement-SuppFig-2.pdf]

Figure S4

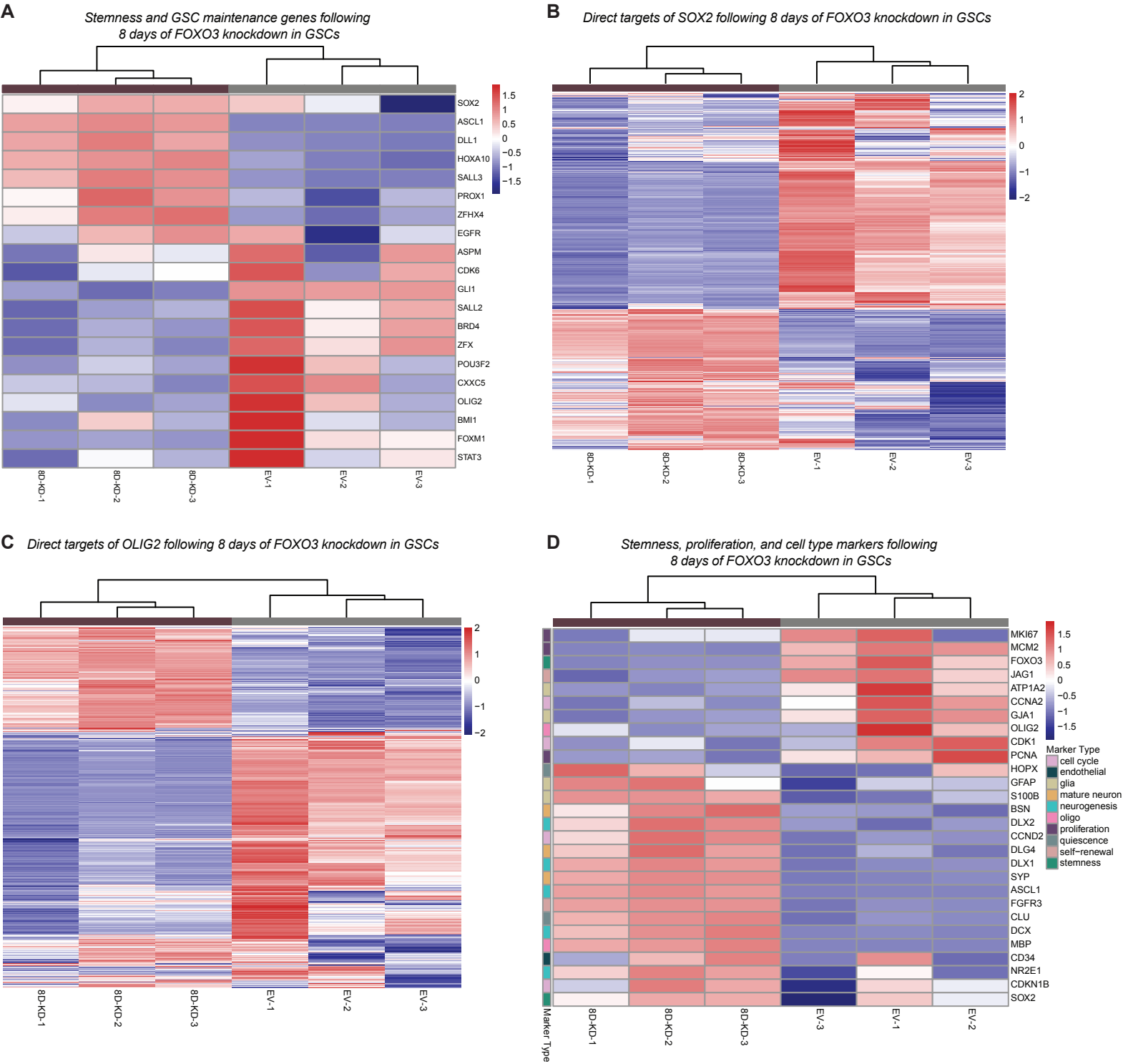

Supplement: SuppFig-4 [file NIHMS1841143-supplement-SuppFig-4.pdf]

Figure S5  
A

Signatures enriched in FOXO3 high and low GSCs

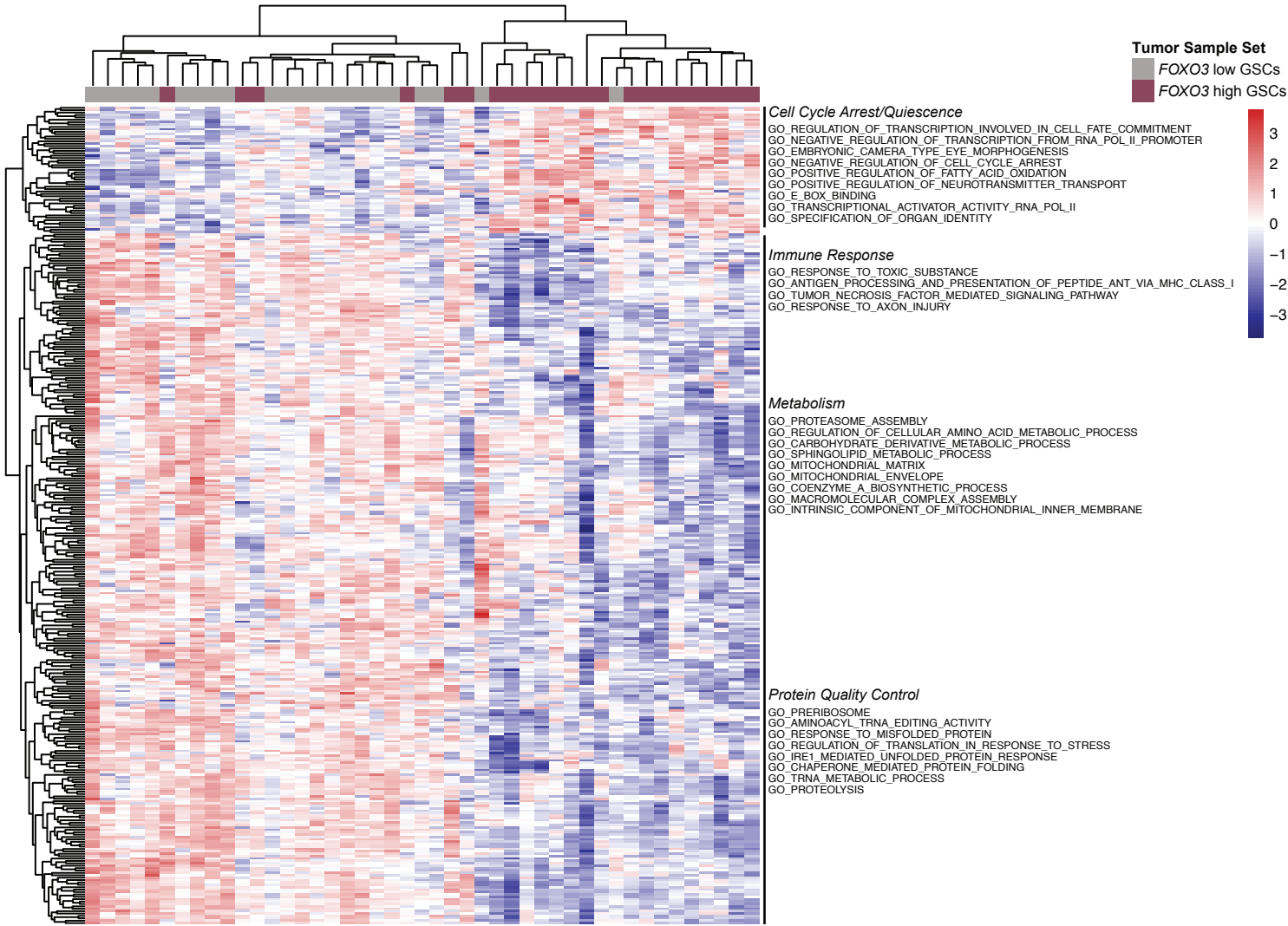

Supplement: SuppFig-5 [file NIHMS1841143-supplement-SuppFig-5.pdf]

Figure S6

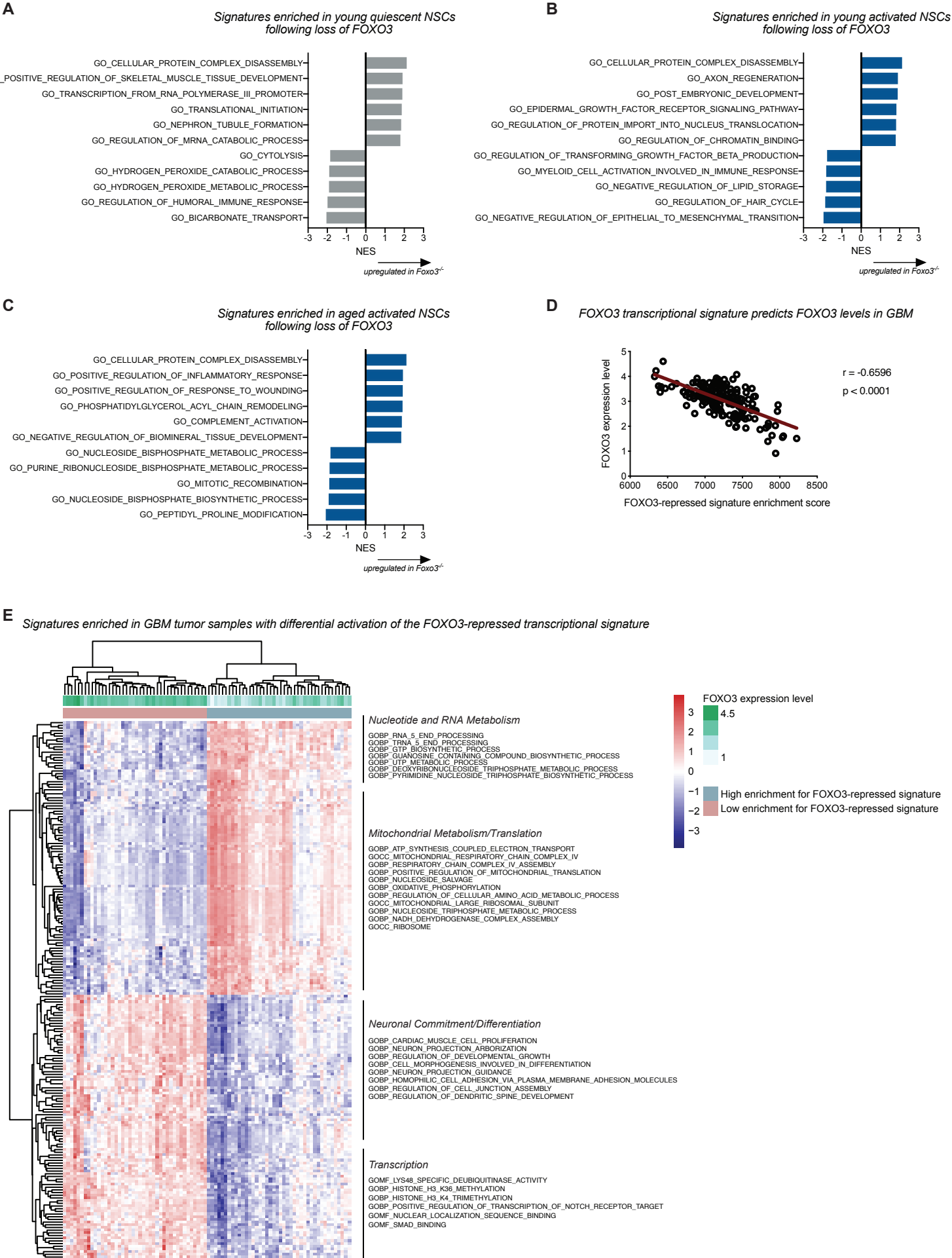

Supplement: SuppFig-6 [file NIHMS1841143-supplement-SuppFig-6.pdf]

Figure S7

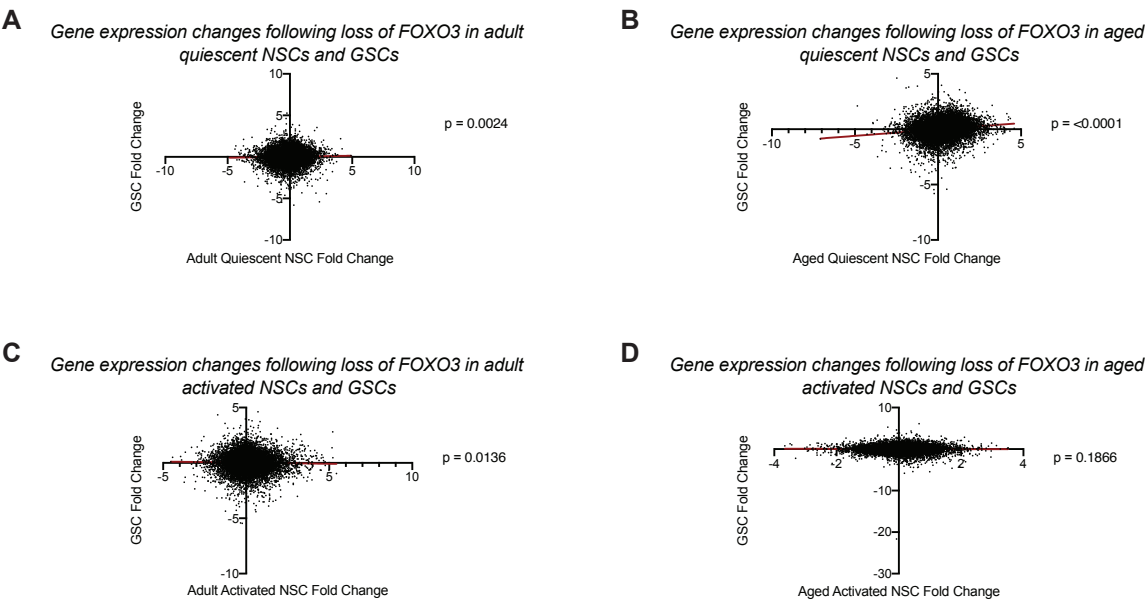

Supplement: SuppFig-7 [file NIHMS1841143-supplement-SuppFig-7.pdf]

Figure S1

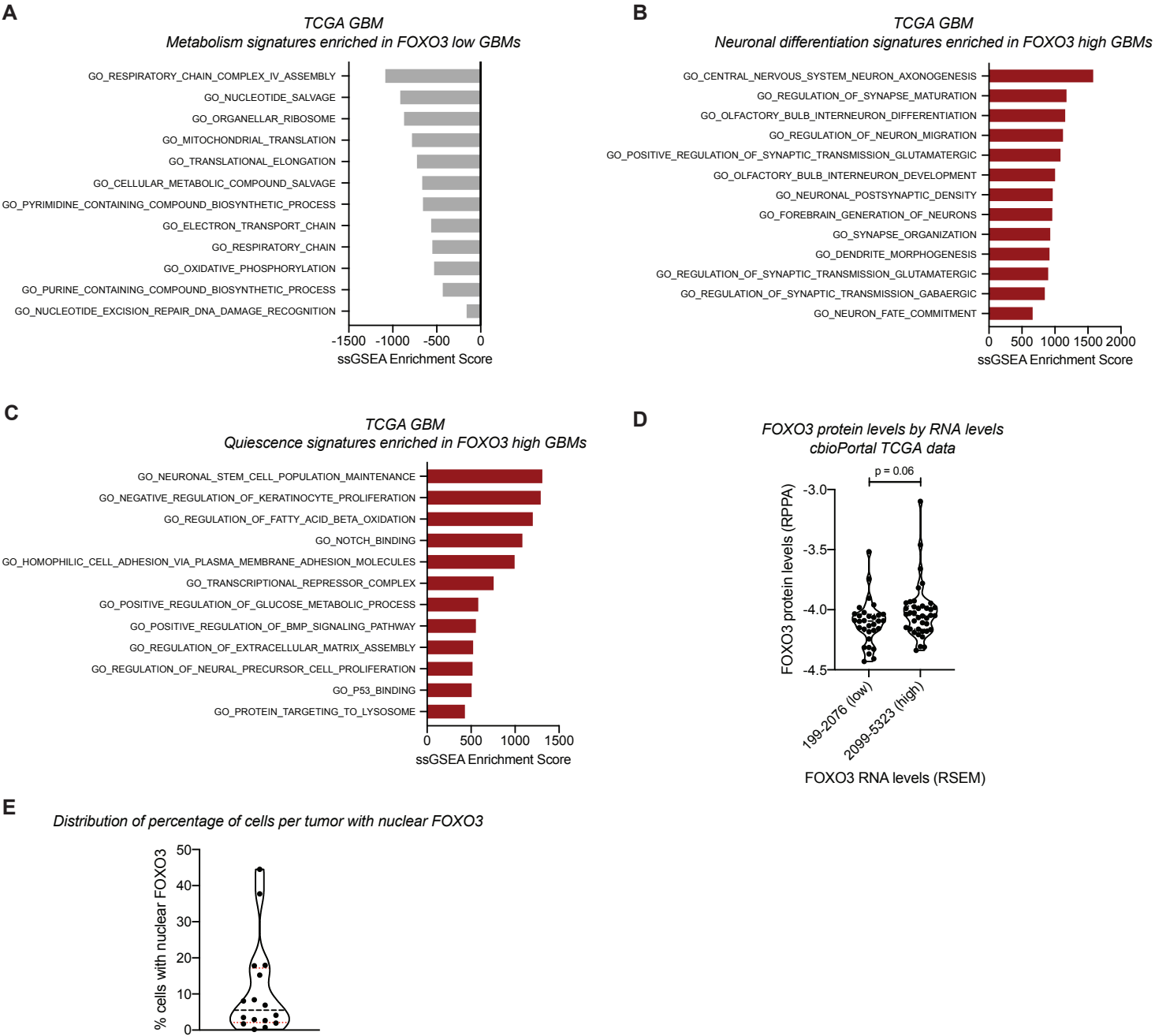

Supplement: SuppFig-1 [file NIHMS1841143-supplement-SuppFig-1.pdf]
