# Supplementary material for: FOXO3 regulates a common genomic program in aging and glioblastoma stem cells
Source: Aging Cancer. Author manuscript; Available in PMC 2022 Oct 26. (PMC9601604; doi:10.1002/aac2.12043)
Supplement: SuppFig-8 [file NIHMS1841143-supplement-SuppFig-8.pdf]

Figure S8

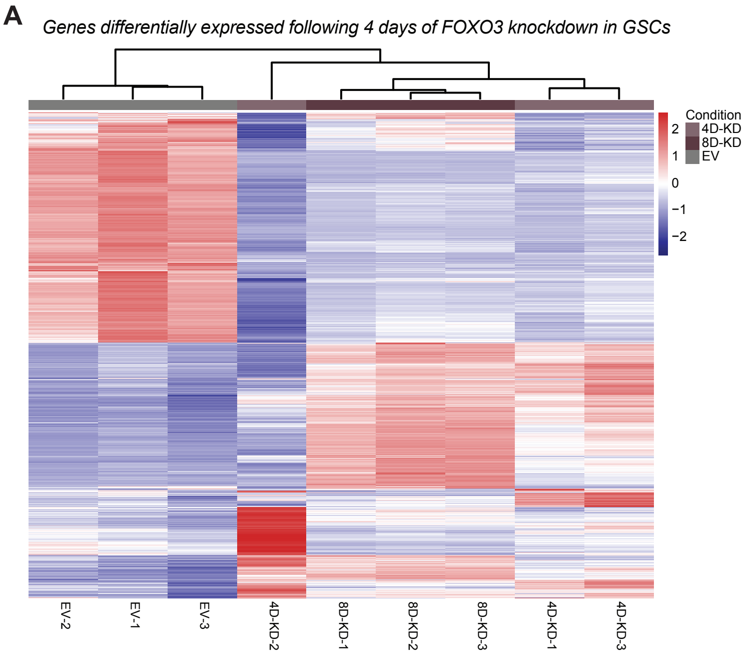
A
Genes differentially expressed following 4 days of FOXO3 knockdown in GSCs
Condition
4D-KD
8D-KD
EV
2
1
0
−1
−2
EV-2
EV-1
EV-3
4D-KD-2
8D-KD-1
8D-KD-2
8D-KD-3
4D-KD-1
4D-KD-3

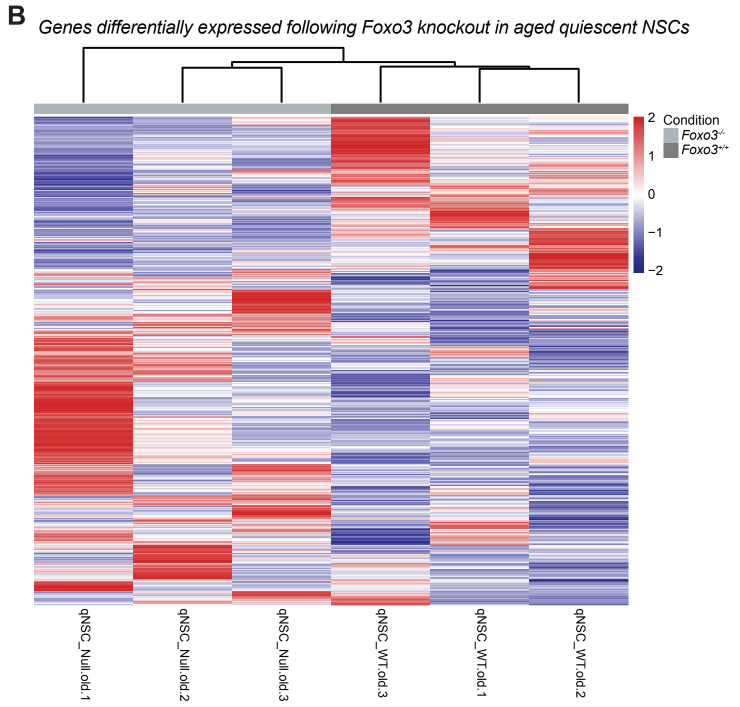
B
Genes differentially expressed following Foxo3 knockout in aged quiescent NSCs
2
1
0
−1
−2
Condition
Foxo3-/-
Foxo3+/+
qNSC_Null.old.1
qNSC_Null.old.2
qNSC_Null.old.3
qNSC_WT.old.3
qNSC_WT.old.1
qNSC_WT.old.2

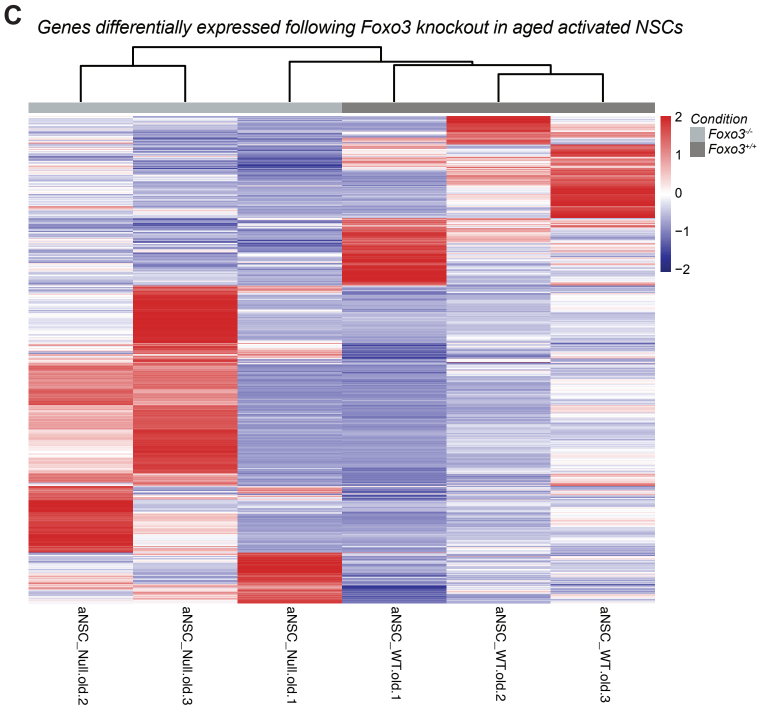
C
Genes differentially expressed following Foxo3 knockout in aged activated NSCs
2
1
0
−1
−2
Condition
Foxo3-/-
Foxo3+/+
aNSC_Null.old.2
aNSC_Null.old.3
aNSC_Null.old.1
aNSC_WT.old.1
aNSC_WT.old.2
aNSC_WT.old.3
