# Supplementary material for: FOXO3 regulates a common genomic program in aging and glioblastoma stem cells
Source: Aging Cancer. Author manuscript; Available in PMC 2022 Oct 26. (PMC9601604; doi:10.1002/aac2.12043)
Supplement: SuppFIg-9 [file NIHMS1841143-supplement-SuppFIg-9.pdf]

Figure S9

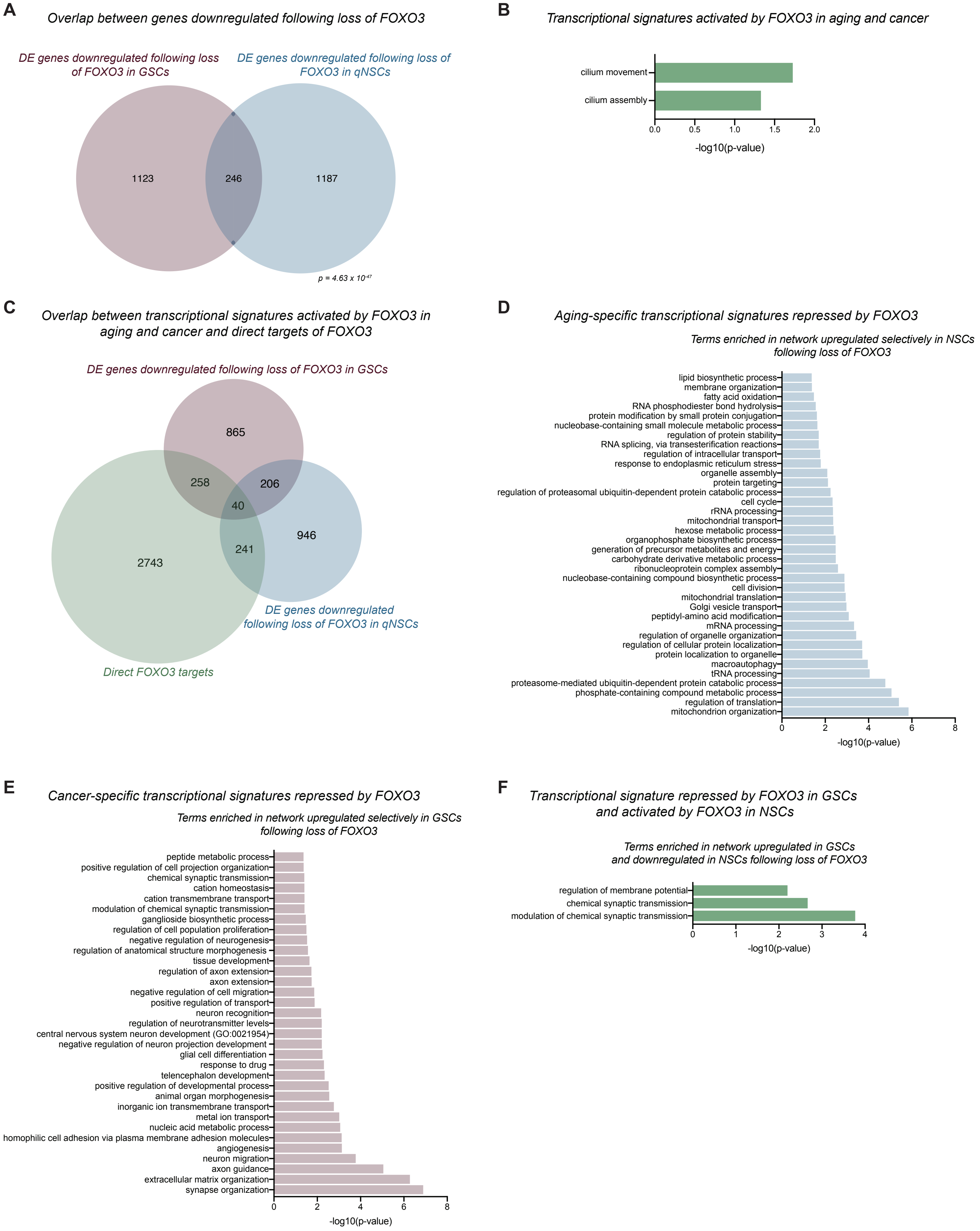
A
Overlap between genes downregulated following loss of FOXO3
DE genes downregulated following loss of FOXO3 in GSCs
DE genes downregulated following loss of FOXO3 in qNSCs
1123
246
1187
p = 4.63 x 10-47
B
Transcriptional signatures activated by FOXO3 in aging and cancer
cilium movement
cilium assembly
0.0
0.5
1.0
1.5
2.0
-log10(p-value)
C
Overlap between transcriptional signatures activated by FOXO3 in aging and cancer and direct targets of FOXO3
DE genes downregulated following loss of FOXO3 in GSCs
865
258
206
40
2743
241
946
DE genes downregulated following loss of FOXO3 in qNSCs
Direct FOXO3 targets
D
Aging-specific transcriptional signatures repressed by FOXO3
Terms enriched in network upregulated selectively in NSCs following loss of FOXO3
lipid biosynthetic process
membrane organization
fatty acid oxidation
RNA phosphodiester bond hydrolysis
protein modification by small protein conjugation
nucleobase-containing small molecule metabolic process
regulation of protein stability
RNA splicing, via transesterification reactions
regulation of intracellular transport
response to endoplasmic reticulum stress
organelle assembly
protein targeting
regulation of proteasomal ubiquitin-dependent protein catabolic process
cell cycle
rRNA processing
mitochondrial transport
hexose metabolic process
organophosphate biosynthetic process
generation of precursor metabolites and energy
carbohydrate derivative metabolic process
ribonucleoprotein complex assembly
nucleobase-containing compound biosynthetic process
cell division
mitochondrial translation
Golgi vesicle transport
peptidyl-amino acid modification
mRNA processing
regulation of organelle organization
regulation of cellular protein localization
protein localization to organelle
macroautophagy
tRNA processing
proteasome-mediated ubiquitin-dependent protein catabolic process
phosphate-containing compound metabolic process
regulation of translation
mitochondrion organization
0
2
4
6
8
-log10(p-value)
E
Cancer-specific transcriptional signatures repressed by FOXO3
Terms enriched in network upregulated selectively in GSCs following loss of FOXO3
peptide metabolic process
positive regulation of cell projection organization
chemical synaptic transmission
cation homeostasis
cation transmembrane transport
modulation of chemical synaptic transmission
ganglioside biosynthetic process
regulation of cell population proliferation
negative regulation of neurogenesis
regulation of anatomical structure morphogenesis
tissue development
regulation of axon extension
axon extension
negative regulation of cell migration
positive regulation of transport
neuron recognition
regulation of neurotransmitter levels
central nervous system neuron development (GO:0021954)
negative regulation of neuron projection development
glial cell differentiation
response to drug
telencephalon development
positive regulation of developmental process
animal organ morphogenesis
inorganic ion transmembrane transport
metal ion transport
nucleic acid metabolic process
homophilic cell adhesion via plasma membrane adhesion molecules
angiogenesis
neuron migration
axon guidance
extracellular matrix organization
synapse organization
0
2
4
6
8
-log10(p-value)
F
Transcriptional signature repressed by FOXO3 in GSCs and activated by FOXO3 in NSCs
Terms enriched in network upregulated in GSCs and downregulated in NSCs following loss of FOXO3
regulation of membrane potential
chemical synaptic transmission
modulation of chemical synaptic transmission
0
1
2
3
4
-log10(p-value)
